# Long-term diet-induced obesity does not lead to learning and memory impairment in adult mice

**DOI:** 10.1371/journal.pone.0257921

**Published:** 2021-09-29

**Authors:** Judith Leyh, Karsten Winter, Madlen Reinicke, Uta Ceglarek, Ingo Bechmann, Julia Landmann

**Affiliations:** 1 Institute of Anatomy, Faculty of Medicine, University of Leipzig, Leipzig, Germany; 2 Institute of Laboratory Medicine, Clinical Chemistry and Molecular Diagnostics, Faculty of Medicine, University of Leipzig, Leipzig, Germany; 3 Leipzig Research Center for Civilization Diseases (LIFE), Faculty of Medicine, University of Leipzig, Leipzig, Germany; Nathan S Kline Institute, UNITED STATES

## Abstract

Obesity arising from excessive dietary fat intake is a risk factor for cognitive decline, dementia and neurodegenerative diseases, including Alzheimer’s disease. Here, we studied the effect of long-term high-fat diet (HFD) (24 weeks) and return to normal diet (ND) on behavioral features, microglia and neurons in adult male C57BL/6J mice. Consequences of HFD-induced obesity and dietary changes on general health (coat appearance, presence of vibrissae), sensory and motor reflexes, learning and memory were assessed by applying a phenotypic assessment protocol, the Y maze and Morris Water Maze test. Neurons and microglia were histologically analyzed within the mediobasal hypothalamus, hippocampus and frontal motor cortex after long-term HFD and change of diet. Long periods of HFD caused general health issues (coat alterations, loss of vibrissae), but did not affect sensory and motor reflexes, emotional state, memory and learning. Long-term HFD increased the microglial response (increased Iba1 fluorescence intensity, percentage of Iba1-stained area and Iba1 gene expression) within the hypothalamus, but not in the cortex and hippocampus. In neither of these regions, neurodegeneration or intracellular lipid droplet accumulation was observed. The former alterations were reversible in mice whose diet was changed from HFD to ND. Taken together, long periods of excessive dietary fat alone do not cause learning deficits or spatial memory impairment, though HFD-induced obesity may have detrimental consequences for cognitive flexibility. Our data confirm the selective responsiveness of hypothalamic microglia to HFD.

## 1. Introduction

The consumption of a high-fat diet (HFD) and consequent obesity are associated with numerous disorders, including cardiovascular diseases, cancer, and metabolic disturbances such as in glucose metabolism and insulin resistance, as well as accumulation of adipose tissue [1–4]. Further, (diet-induced) obesity has been described to be linked with cognitive deficits and increased risk of neurodegenerative diseases, including Alzheimer’s disease, by exacerbating brain inflammation and accelerating brain aging [5–7].

An important and critical brain region for memory consolidation and recall is the hippocampal formation [8, 9], a structure which has been shown to be very vulnerable to effects of obesity or poor nutrition [10, 11]. Several studies demonstrated that rodents with excessive dietary fat and sugar intake for months are impaired in hippocampal-dependent memory tasks [12–16]. Notably, some feeding studies in adult rodents and humans indicated that even few days of obesity-induced diet is sufficient to affect the function of the hippocampus [10, 17–21]. Yaseen et al. (2019) and Khazen et al. (2019) recently reported that acute exposure to HFD at juvenility in male rats is linked to impaired social recognition memory and compromised prefrontal plasticity, and it is associated with disrupted hippocampal-dependent memory and plasticity [16, 22]. Several studies indicated that adolescent HFD exerts more deleterious effects on hippocampal-dependent plasticity and memory as compared to HFD exposure during adulthood [23–25]. There is also increasing evidence that altered hippocampal function in obesity may impair adaptive decision making around eating and food [7]. In addition, numerous data indicate that HFD may activate signaling pathways with harmful effects not only in the hippocampus, but also in the cortex [26–28].

Apart from the cortex and hippocampus, another crucial brain area being vulnerable to diets high in fat and sugar is the hypothalamus, which plays a pivotal role in controlling appetite and weight within the central nervous system (CNS). Several hypothalamic neuronal populations act as important sensors and regulators of peripheral metabolism and glial cells are involved in body weight homeostasis [29–38]. While astrocytes and oligodendrocytes are primarily involved in neuronal development, survival and function, microglia are the brain’s immunocompetent macrophages [39–41]. They crucially contribute to homeostasis, plasticity and learning by taking up synaptic remnants, toxins and myelin debris [42–44]. Several studies reported that the activation state of microglial cells is affected by obesity [45], western lifestyle and nutrition [46] as well as the microbiome, which provides short-chain fatty acids required for microglial maturation [47]. A study by Valdearcos et al. (2014) in rodents showed that microglia within the mediobasal hypothalamus take up saturated fatty acids triggering inflammatory signaling leading to neuronal dysfunctions, whereas unsaturated fatty acids can even exert anti-inflammatory effects in the hypothalamus [48].

In diet-induced obesity, elevated levels of circulating fatty acids and an increase in secreted pro-inflammatory cytokines, amongst other factors, induce systemic inflammation progressively initiating microglial activation, endothelial damage, and disruption of the blood brain barrier. These events eventually lead to brain inflammation, although to a varying extent depending on numerous factors such as level of obesity, age, diet composition, and CNS structure examined, and gradually result in synaptic loss and neuronal death [49, 50].

In this study, we addressed neuroanatomical alterations and prospective behavioral consequences of long-term HFD on general health, sensory and motor reflexes, learning and memory. Further, we investigated whether long-term HFD impairs neurons and microglia within three brain regions, the hypothalamus, the hippocampus and the cortex, being especially affected by poor nutrition. Additionally, we examined potential benefits after a change from a fat-dense nutrition to ND.

## 2. Materials and methods

### 2.1 Animals and diets

**Fig 1:**
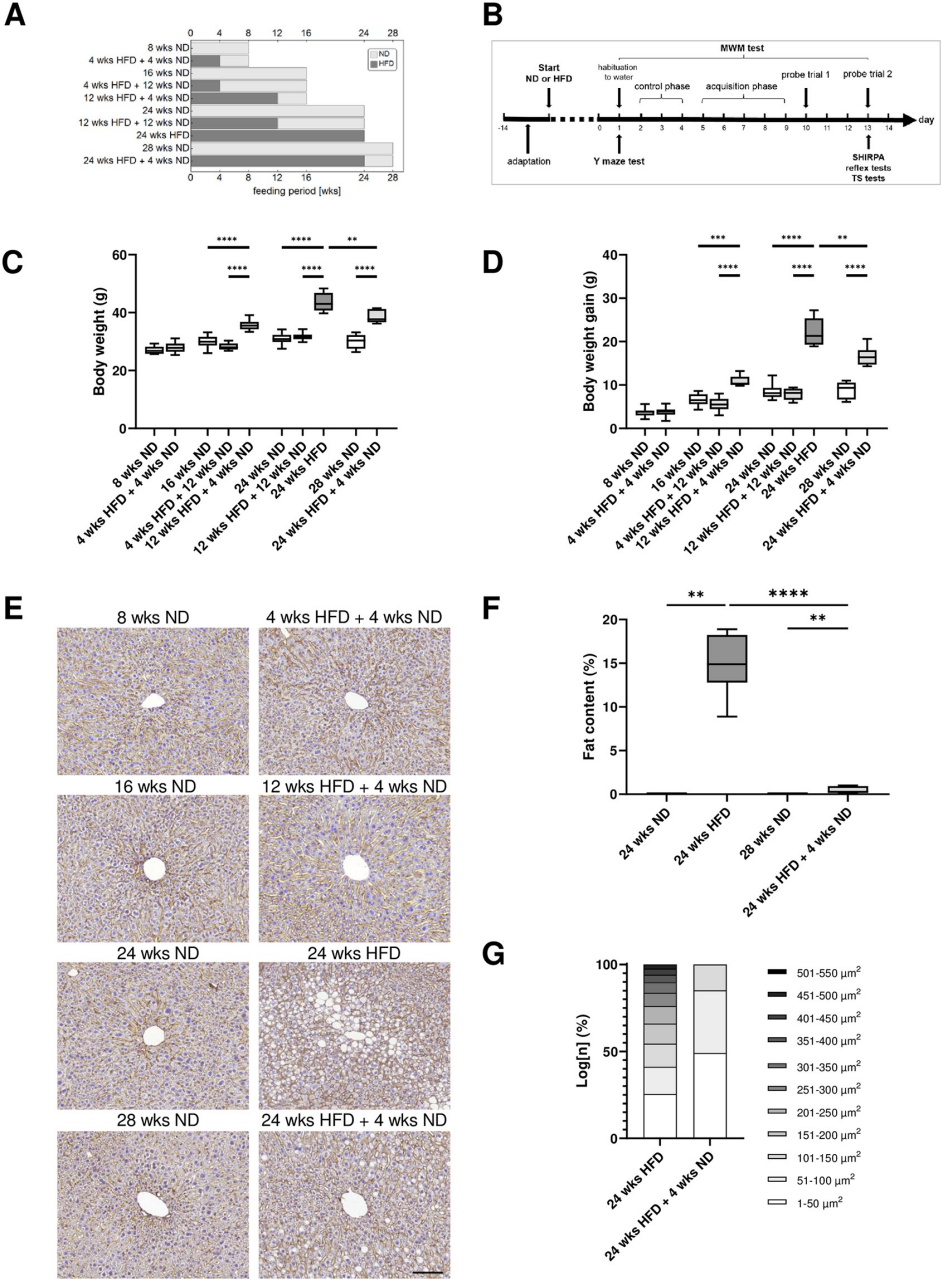
Effect of age, diet and dietary change on body weight and liver tissue. (A) Experimental groups and feeding period. (B) Experimental set-up and sequence of behavioral tests. Mice had 14 days to adapt until diet (ND or HFD) started. Study began with sensitive and cognitive tests (Y maze and MWM test) and terminated with SHIRPA, shorter reflex and tail suspension tests. For histochemical analysis, mice were sacrificed on day 14. (C) Body weight and (D) weight gain of C57BL/6J mice fed with HFD and/or ND for varying wks. (E) Representative liver sections stained for β-catenin and hematoxylin from mice maintained on HFD and/or ND for varying wks, scale bar corresponds to 50 μm. (F) Quantification of percentage of lipids in β-catenin- and hematoxylin-stained liver sections and (G) quantitative analysis of the proportion (grouped according to size) of lipid droplets in hepatocytes revealed a long-term HFD-induced hepatic steatosis in mice. Data are presented as mean values and error bars indicate SD; 8 wks ND n = 11 [5], 4 wks HFD + 4 wks ND n = 12 [5], 16 wks ND n = 24 [6], 4 wks HFD + 12 wks ND n = 12 [6], 12 wks HFD + 4 wks ND n = 8 [6], 24 wks ND n = 30 [12], 12 wks HFD + 12 wks ND n = 12 [6], 24 wks HFD n = 10 [6), 28 wks ND n = 12 [6], 24 wks HFD + 4 wks ND n = 7 [6]; in brackets, the number of animals used for liver analysis; (C, D): One-way ANOVA, Welch-ANOVA, unpaired t test, Kruskal-Wallis test; (F): Mann-Whitney test, unpaired t test; **** p < 0.0001; *** p < 0.001; ** p < 0.01.

The experiments were performed using male wild-type C57BL/6J mice, which were kept in the local animal facility under standard conditions: 12 h dark/light cycle, group-housed with free access to water and food. Young adult male mice (8 weeks (wks) old) were fed with a normal diet (ND) (11 kcal% fat, 53 kcal% carbohydrates, 36 kcal% protein; V1124-300, ssniff Spezialdiäten, Soest, Germany) for 8, 16, 24, and 28 wks or a high-fat diet (HFD) (59 kcal% fat, 26 kcal% carbohydrates, 15 kcal% protein; E15772-34, ssniff Spezialdiäten GmbH, Soest, Germany) for 24 wks or received a dietary change back to ND for 4 or 12 wks after different periods of HFD (4, 12, 24 wks). Laboratory animals were divided into the following ten groups: 8 wks ND (n = 12), 4 wks HFD + 4 wks ND (n = 12, one animal died before the probe trial two of the MWM has been conducted), 16 wks ND (n = 24), 4 wks HFD + 12 wks ND (n = 12), 12 wks HFD + 4 wks ND (n = 12 each), 24 wks ND (n = 18), 12 wks HFD + 12 wks ND (n = 12), 24 wks HFD (n = 18), 28 wks ND (n = 12), 24 wks HFD + 4 wks ND (n = 12) (Fig 1A). Body weight was measured weekly during the whole experiment and daily during behavioral testing. For analysis of the consequences of diet or dietary change we set the following thresholds for a minimal weight gain compared to ND: 25% for 24 wks HFD, 20% for 24 wks HFD + 4 wks ND and 10% for 12 wks HFD + 4 wks ND. Mice which did not reach the threshold were excluded from analysis. All animal experiments were approved by the local state and university authorities. We performed this study in accordance with the guidelines of the Animal Experimental Committee following the German Animal Welfare Act as well as the European guidelines (Directive 2010/63/EU) concerning the protection of laboratory animals. All experimental procedures and protocols were authorized by the local ethics committee of the state of Saxony (Landesdirektion Sachsen, Leipzig, approval no. TVV 41/17).

### 2.2 Behavioral phenotyping

Behavioral testing was accomplished in a silent, separate room (dim illumination, constant temperature (23°C) and humidity) during the active phase of the animals. Mice had one hour to adjust to the testing room before experiments started. Tests were video-recorded and analyzed later using Video Mot2 software or evaluated and scored directly by an experienced researcher. All behavioral tests were conducted and analyzed by the same experimenter blinded to conditions. After each trial, all testing devices were cleaned with 70% ethanol. An overview of behavioral tests is illustrated in Fig 1B. Memory tests were performed first, followed by tests that required repeated handling. All animals and groups mentioned in 2.1 passed through all behavioral tests described below.

#### Y maze test

We investigated working and spatial memory following HFD or dietary change using a three-armed horizontal maze (each arm 5 cm x 25 cm) made of white opaque Plexiglas. Test details can be found in Landmann et al. (2019) [51]. Mice were placed in the center of the testing arena and had the liberty to explore the environment freely over 7 min, while the trial was video-recorded from above. Videos were converted to images using FFmpeg (Version 3.3.9, https://ffmpeg.org) and image processing was performed using Mathematica (Version 11.3, Wolfram Research Inc., Champaign, IL, USA). Mice were automatically detected. Based on all mouse positions the Y maze shape was reconstructed, all three arm end points as well as the central junction were automatically detected and the three maze arms were labeled. Mouse positions were assigned according to the labeled arms and respective sequential durations of stay of the mouse within the arms were calculated based on the frame rate. The percentage of spontaneous alternations between arms was calculated as follows: [number of alternations/ (total number of arm entries) − 2)] * 100 [52].

#### Morris Water Maze test

Applying the Morris Water Maze test (MWM) we studied learning and long-term memory adapted from previous studies [53, 54]. The set-up consisted of a swimming pool made of white opaque Plexiglas (diameter 120 cm, height 60 cm) with a removable transparent platform (diameter 10 cm, height 20 cm, 1 cm below the water surface). The water temperature was kept constantly at 23–24°C. Water was made opaque by non-toxic white water color. The testing area was separated from the experimenters’ location and the other animals by an opaque folding screen. Animals in the swimming pool were video-recorded from above and tracked automatically (Video Mot2). Initially, we performed a habituation and a control phase to evaluate comparability of data due to visual and motoric capabilities between young and old as well as slim and obese mice (Fig 1B). After the Y maze test, mice were placed three times for 10 sec each onto the platform in the swimming pool for habituation to the water. If the mouse jumped into the water, the animal was carefully guided back to the platform. In the control session (three days), the location of the platform hidden underneath the water surface was indicated by a high contrast check mark. In the following acquisition phase over four days, mice should find the hidden platform using visual cues at the border of the basin. Each mouse was tested in three trials (max. 60 sec each) and was placed under red light between the trials. At day 10, after the training phase, memory retention was tested in probe trial one, where the platform was removed. After two days without training mice were tested again in probe trial two (Fig 1B). Fecal pellets and odors were removed from the water after each trial and water was changed daily.

#### SHIRPA

We followed a modified SHIRPA protocol (SmithKline Beecham, Harwell, Imperial College, Royal London Hospital, phenotype assessment) to assess general health of mice after different types and periods of nutrition. Mice’s appearance and behavior were evaluated using following parameters and scores: palpebral closure (0 = eyes open; 1 = eyes closed), coat appearance (0 = tidy, well groomed; 1 = shaggy, dirty), skin color (0 = blanched, 1 = pink, 2 = deep red), whiskers (0 = absent, 1 = present), tail elevation (0 = dragging, 1 = horizontal extension, 2 = elevated/straub tail), body position (0 = inactive, 1 = active, 2 = excessively active), head tremble (0 = no reaction, 1 = mild shaking, 2 = strong shaking), gait (0 = fluid movement, 1 = irregular, anomalies), touch escape (0 = no reaction, 1 = reaction to touch, 2 = strong reaction to touch/jumps or flees prior), lacrimation (0 = absent, 1 = present), defecation (0 = absent, 1 = present), vocalization (0 = none, 1 = vocal) and biting (0 = none, 1 = present in response to handling). In order to evaluate the effect of diet or dietary change, we measured the body weight.

#### General reflexes

General sensory and motor abilities were estimated by the following reflexes: vibrissae reflex (0 = no reaction, 1 = reaction to whisker touch, 2 = strong reaction to whisker touch), pinna reflex (0 = no reaction, 1 = reaction to pinna touch, 2 = strong reaction to pinna touch) and writhe reflex (0 = absent, 1 = present) [51].

#### Tail suspension test

As described by Cryan et al. (2005), mice were suspended by the tail and video-recorded for three min to analyze their behavior to escape (learned helplessness) [55]. Thereby, we evaluated how emotionally stable and stress-resistant animals were, which could influence their performance in cognitive tasks. Time and latency spending active or inactive were measured. Each mouse was tested once and placed back in the home cage at the end. Video processing was conducted exactly as described for the Y maze experiments. Mice were automatically detected and change in segmented mouse size between consecutive frames was considered as an activity marker. Phases of activity and inactivity were automatically detected and durations were calculated based on the frame rate.

### 2.3 Tissue preparation

At the end of the feeding experiments mice were anesthetized with isoflurane (Baxter GmbH, Unterschleißheim, Germany) and transcardially perfused with ice-cold phosphate buffered saline (PBS, pH 7.4) and 4% paraformaldehyde (PFA) in 0.2 M PBS. Brains were carefully removed from the skull and post-fixed for 24 hours in 4% PFA in 0.2 M PBS before their storage in PBS, containing 0.2% sodium azide, until further processing. Livers of mice were collected for further analysis.

### 2.4 Histopathological assessment of liver tissue

Liver tissue was fixed with 4% PFA in 0.2 M PBS, dehydrated in graded ethanol series, dealcoholized in xylene and paraffin embedded. Paraffin blocks were sliced into five μm sections, stained with mouse anti-β-catenin (1:500; BD Transduction Laboratories, Franklin Lakes, New Jersey, USA) and counterstained with hematoxylin solution.

Whole β-catenin-stained sections were fully digitized at 20x magnification using a digital slide scanner (Pannoramic Scan II, 3D HISTECH Ltd., Budapest, Hungary). The scanner software (Pannoramic Scanner, version 1.23, 3D HISTECH Ltd., Budapest, Hungary) was operated in extended focus mode (three levels with 0.8 μm axial distance) to combine images from several adjacent focal planes into one image with maximum depth of sharpness. Regions of interest (ROIs) within the specimen were exported from slide scanner data sets (CaseViewer, Version 2.3, 3D HISTECH Ldt., Budapest, Hungary) as PNG images with pixel dimensions of 0.243 μm.

For quantification of hepatic lipid accumulation, PNG images were imported into Mathematica (Version 12.0, Wolfram Research Inc., Champaign, IL, USA) and tissue masks were computed using global thresholding (t = 0.7). These masks contained the tissue itself along with holes representing small and/or scattered lipid accumulations, fully formed vacuoles, vessels as well as artifacts like cuts, fissures or incisions. Almost all lipid accumulations and vacuoles could be detected automatically by using empirically determined size thresholds (200 < t < 4000) as well as a roundness parameter (bounding disk coverage > 0.53) for the characterization of these holes. Masks of all remaining structures were superimposed onto the original images and these automatically generated pre-selections were inspected and corrected by hand using GIMP (Version 2.10.2, The GIMP team, http://www.gimp.org). Total tissue area and area of lipid accumulations or vacuoles were counted, and ratios were calculated. Furthermore, areas of all individual lipid accumulations and vacuoles were calculated and averaged per image.

### 2.5 Fluorescence labeling

For immunofluorescence staining with rabbit anti-Iba1 (ionized calcium-binding adaptor molecule 1; Synaptic Systems, Göttingen, Germany) to label microglia and guinea pig anti-NeuN (Synaptic Systems, Göttingen, Germany) to label neurons, perfused and fixed mouse brains were sliced into 50 μm thick coronal floating sections using a vibratome (Leica VT 1200, Leica Biosystems, Wetzlar, Germany). After three washing steps with 0.3% Triton X-100 in PBS for 10 min each time, slices were blocked for one hour in PBS blocking buffer containing 5% normal goat serum and 0.3% Triton X-100 at room temperature. Afterwards, coronal brain sections were incubated with the primary antibodies Iba1 (1:500) and NeuN (1:200) diluted in PBS with 1% of normal goat serum. Incubation was done overnight at 4°C. The next day, slices were rinsed three times with 0.3% Triton X-100 in PBS and incubated with the secondary antibodies goat anti-guinea pig Alexa Fluor 488 (1:200) (Thermo Fisher Scientific, Waltham, Massachusetts, USA) and goat anti-rabbit Alexa Fluor 568 (1:250) (Thermo Fisher Scientific) for 2 hours at room temperature. Thereafter, sections were washed with PBS, stained five min with 4′,6-diamidino-2-phenylindole (DAPI; Thermo Fisher Scientific) and were thoroughly rinsed in PBS. Finally, brain sections were mounted onto microscope slides and covered with Fluorescence Mounting Medium (DAKO, Agilent, Santa Clara, California, USA) and coverslips. For negative controls, the omission of primary antibodies, under otherwise identical conditions, resulted in the absence of any labeling.

### 2.6 Oil Red O staining

Perfused and fixed mouse brains were sliced with a vibratome (Leica VT 1200, Leica Biosystems, Wetzlar, Germany) into 20 μm thick coronal floating sections for Oil Red O Staining. Brain sections were washed three times with PBS for five min each time followed by incubation with 60% isopropanol for five min. Thereafter, slices were stained with Oil Red O solution [60% Oil Red O stock solution (5 mg/ml isopropanol)/40% water] for 15 min and washed once again three times with PBS for five min each time. Following a short incubation with 40% isopropanol, brain slices were thoroughly rinsed in PBS and counterstained with hematoxylin solution for one min. Finally, brain sections were mounted onto microscope slides and covered with Glycergel mounting medium (DAKO, Agilent, Santa Clara, California, USA) and coverslips.

### 2.7 Image acquisition and quantification of fluorescence staining

Microscopic images of Iba1 and NeuN staining were captured with a confocal microscope (LSM 700, Zeiss, Jena, Germany) applying a 20×/0.5 NA objective at constant exposure times within hypothalamic, hippocampal and neocortical regions. Confocal z-stack images were acquired using the ZEN 2 (blue edition) software (Zeiss) and an interval size of 2.0 μm for a total range of 30 μm (n = 16 optical slices per animal, 6–12 animals per condition). All in all, five ROIs, the mediobasal hypothalamus, the CA1 and CA3 region, the dentate gyrus, and the frontal motor cortex were acquired for each animal to quantify Iba1 and NeuN immunosignals. Two ROIs per animal within the mentioned brain areas were analyzed in coronal brain sections at Bregma -1.7 mm. Fluorescence intensity and staining area measurements of z-stack maximum intensity projections were processed using ImageJ software (National Institutes of Health, Bethesda, MD, USA). The percent area occupied by Iba1- or NeuN-immunopositive cells per ROI was measured after a threshold adjustment of the images. The total staining intensity was expressed by integrated density (mean gray value x area) using the ROI manager and the background subtraction function of ImageJ. The reactive state of microglial cells is frequently measured by fluorescence intensity of Iba1 or by percentage of Iba1-stained area [37, 45, 56–59].

For quantitative analysis of microglial morphology, original confocal z-stack images were imported into Mathematica and maximum intensity projections of the Iba1 channels were computed. Microglia cells were automatically detected using a previously developed approach [60]. In brief, images were contrast enhanced, Iba1-positive structures (somata and processes) were segmented and all somata were identified. Processes not connected to any somata were removed and all interconnected microglia cells were separated from each other based on a parallel flood fill operation starting from the soma centroids. ROIs were drawn onto the maximum intensity projections and used for the masking of all detected cells. Remaining cells within the respective regions were uniquely labelled. Further, microglial cells were submitted to quantification and 17 parameters were calculated for each detected Iba1-positive cell (n = 9186). Parameters include *cell areas* (μm^2^) and *perimeters* (μm) of whole cells, their *convex hulls* (the smallest convex set of pixels that encloses a cell) and their soma; *cell solidity* (the degree to which the *area* of a cell fills the area of its *convex hull*) and *convexity* (the ratio of a cell’s *convex hull perimeter* to the cell’s actual perimeter); *circularity* of cells and soma (the roundness, where one equals a perfect circle and values smaller than one indicate shapes that increasingly deviate from the shape of a circle); *length* (μm) as well as number of *branch* and *end points* of the skeletonized *processes*; and the number of *cell processes*. Furthermore, all cells were submitted to Sholl analysis [61] and the cell’s *branching index* [62] (a measure to distinguish between cells of different ramification types), *critical radius* (μm) (the radius with the maximum number of process crossings) and *dendritic maximum* (the number of process crossings at the critical radius) were calculated. Values for each parameter were averaged for all images of one animal to define the value per animal and per group, respectively. Additionally, for whole images the microglial cell density (cells per mm^2^) was computed.

### 2.8 Image acquisition and quantification of Oil Red O staining

The staining of lipid droplets in coronal brain sections using Oil Red O solution was fully digitized using the same digital slide scanner and image export procedures mentioned above (see 2.4). The only exception was that the extended focus mode was set to 30 levels with 1.2 μm axial distance at 40x magnification to combine images from several adjacent focal planes into one image with maximum depth of sharpness resulting in exported PNG images with pixel dimensions of 0.122 μm.

For quantification of lipid droplets, PNG images were imported into Mathematica, white-balanced and submitted to color deconvolution resulting in separate images for red (Oil Red O) and blue (hematoxylin) staining. Red images were background-corrected (ten pixel wide Gaussian filter), lipid droplets were segmented using local adaptive thresholding (100 pixel wide radius) and specks smaller than ten pixels were removed. Blue images were adjusted for brightness and gamma, nuclei were segmented using global thresholding (threshold value 0.15) followed by morphological closing (three pixel radius), and specks smaller than 200 pixels were removed. Due to the varying quality of Oil Red O staining segmented droplets were manually corrected using GIMP to remove tissue artifacts when necessary. Total tissue area, cell area, droplet area and red signal intensity were counted and ratios were calculated.

### 2.9 Quantitative RT-qPCR

Hypothalamus, hippocampus and frontal cortex were quickly dissected from the brain of mice fed with ND or HFD for 24 wks after transcardial perfusion with ice-cold phosphate buffered saline (PBS, pH 7.4). Brain tissue was flash-frozen in liquid nitrogen and stored at −80°C until RNA isolation.

RNA isolation and cDNA synthesis: Messenger RNA (mRNA) of the hypothalamus, hippocampus and prefrontal murine cortices was isolated using RNeasy Mini Kit (Qiagen, Hilden, Germany) according to manufacturer’s instructions. Reverse transcription was performed with the ProtoScript First Strand Synthesis Kit (New England Biolabs, Frankfurt am Main, Germany) using 1 μg total RNA as template.

RT2 Profiler^™^ PCR array: Gene expression of *Iba1* was analyzed using a RT2 Profiler^™^ PCR array. Primers were synthesized by the manufacturer Qiagen and are adsorbed on the bottom of each well in a 96-well microplate, one primer pair per well. Each PCR array plate includes three housekeeping genes (*Actb*, *Gapdh*, *B2m*) as well as controls for genomic DNA contamination, reverse transcription efficiency and general PCR performance. Thermal cycling and fluorescence detection were performed using the CFX96 Touch Real-Time PCR Detection System from Bio-Rad Laboratories GmbH (Feldkirchen, Germany). The utilized temperature protocol includes an initial melting for 10 min at 95°C, 40 cycles of amplification (15 s at 95°C, 1 min at 60°C) followed by a melt curve. Relative gene expression was calculated using the ΔΔCt method (2-ΔΔCt). All Ct values of target cDNAs were normalized to the average of three housekeeping genes.

### 2.10 Statistical analysis

Behavioral data were tested for normal distribution using the D’Agostino & Pearson test. Nonparametric data (scored parameters or parameters not following a normal distribution) were analyzed by the Kruskal-Wallis test followed by Dunn’s method for multiple comparisons or the Mann-Whitney test. For parametric data (following a normal distribution) one-way ANOVA and unpaired t test (equal SDs) or Welch-ANOVA and Welch’s t test (unequal SDs), and for multifactorial data two-way ANOVA were performed using Tukey’s method for multiple comparisons. Immunohistochemical data of liver tissue were tested for normal distribution using the Kolmogorov-Smirnov test and validated by Mann-Whitney (non-parametric) or unpaired t test (parametric). Data of immunofluorescence staining were tested for normal distribution using the Shapiro-Wilk test and were analyzed by one-way ANOVA followed by Tukey’s method for multiple comparisons, unpaired t test or Welch-ANOVA followed by Dunnett’s method for multiple comparisons. The qPCR data was tested for normal distribution using the Shapiro-Wilk test and differences between the ND and HFD group were validated by an unpaired t test. Analysis of data was performed separately within age-matched (4 sub-analyses: 8 wks ND vs. 4 wks HFD + 4 wks ND; 16 wks ND vs. 4 wks HFD + 12 wks ND vs. 12 wks HFD + 4 wks ND; 24 wks ND vs. 12 wks HFD + 12 wks ND vs. 24 wks HFD; 28 wks ND vs. 24 wks HFD + 4 wks ND) as well as diet-matched (3 sub-analyses: 8 vs. 16 vs. 24 vs. 28 wks ND; 4 wks HFD + 4 wks ND vs. 12 wks HFD + 4 wks ND vs. 24 wks HFD + 4 wks ND; 4 wks HFD + 12 wks ND vs. 12 wks HFD + 12 wks ND) groups. We compared age-matched mice to analyze the impact of diet and dietary change as well as diet-matched mice to examine the effect of age on behavioral features, microglia and neurons. For better clarity, only significant results of age-matched groups are presented in the figures and significant results of diet-matched groups are shown in S1 Table. GraphPad Prism 9.2.0 (GraphPad Software, San Diego, CA, USA) was applied to perform statistical analyses of behavioral and histological data. Statistic details are given in the figure legends, result section and S1 Table. Number of analyzed animals is indicated as “n” in the figure legend. Data are presented as mean ± SD. Statistical significance was determined as follows: p < 0.05 *, p < 0.01 **, p < 0.001 ***, p < 0.0001 ****.

## 3. Results

### 3.1 HFD and dietary change have an effect on body weight and liver tissue

As expected, diet had a highly significant effect on mice’s body weight and animals benefited significantly from a dietary change back to ND after HFD exposure (Fig 1C; S1 Table, ll. 1–3). Thereby, even a short period of four wks ND following a long-term HFD of 24 wks revealed a positive effect on the body weight (Fig 1C; unpaired t test, p = 0.0024; S1 Table, l. 4). Further, we observed a strong effect of age on body weight as well (S1 Table, ll. 5–7). Older mice fed with ND gained significantly more body weight compared to younger animals (Fig 1D; S1 Table, l. 12).

To control the effectiveness of HFD, we performed staining of liver tissue with β-catenin and hematoxylin and found an increase in lipid accumulation after long-term HFD compared to mice fed with ND (Fig 1E and 1F; Mann-Whitney test, p = 0.0022; S1 Table, l. 15) as shown before [63, 64]. Mouse liver tissue in ND groups and groups that received short- and mid-term HFD (four and 12 wks) followed by a dietary change back to ND exhibited the typical hepatolobular architecture and hepatocytes displayed their normal polygonal shape showing distinctive nuclei along with no signs of steatosis (Fig 1E). In contrast, we observed high amounts of lipid droplets (macrovesicular steatosis) after long-term HFD in liver tissue. However, already four wks ND after 24 wks HFD significantly improved the livers’ fat content, but these mice exhibited even more lipid droplets in hepatocytes than their age-matched control group (Fig 1E and 1F; unpaired t test, p < 0.0001, Mann-Whitney test, p < 0.0022; S1 Table, ll. 16–17). Moreover, lipid droplet sizes showed a shift of hepatic lipid droplets toward smaller ones in mice that received long-term HFD followed by a dietary change. Long-term HFD alone also leads to hepatic lipid droplets of enormous size (Fig 1G).

### 3.2 Long periods of HFD lead to general health issues without modification of reflexes

**Fig 2:**
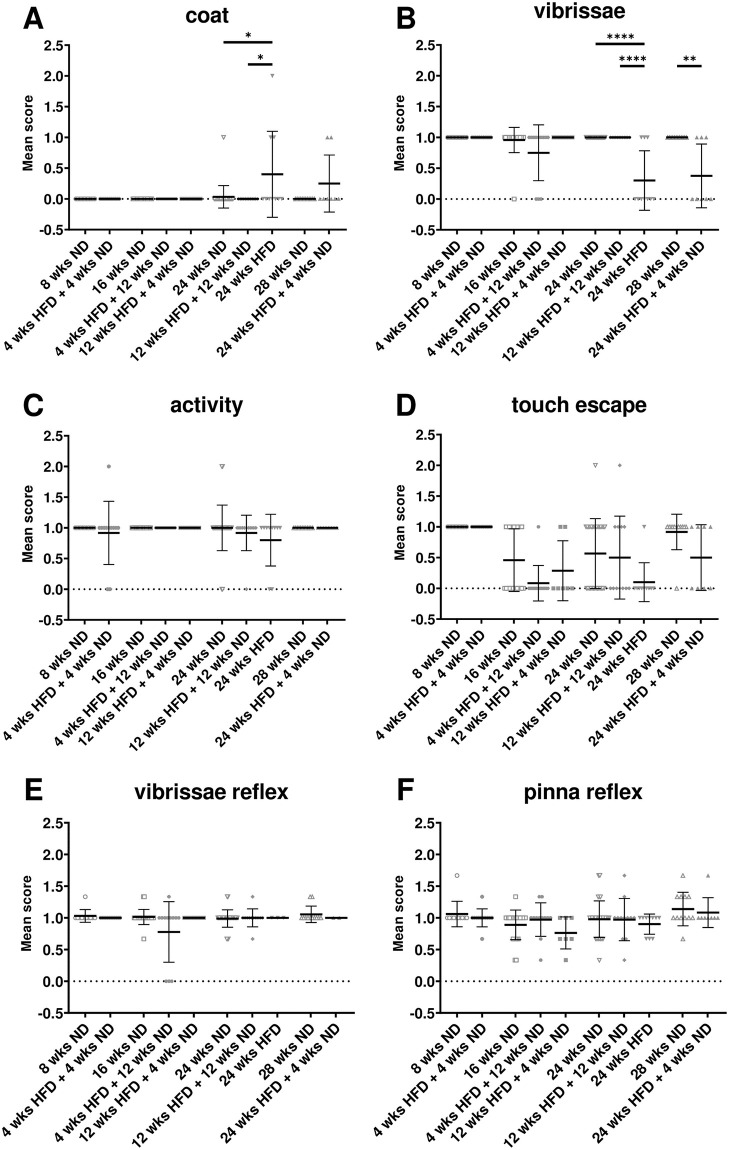
HFD leads to general health issues. (A-D) SHIRPA analysis was used to estimate general health of mice after ND or HFD. (A) Long-term HFD led to problems with the coat and (B) the loss of vibrissae, while there was more variance, but no significant difference after HFD in (C) general activity and (D) touch escape. Normal sensory reflexes as the (E) vibrissae and the (F) pinna reflex were observed in all groups irrespective of diet or age. Data are presented as mean values and error bars indicate SD; 8 wks ND n = 11, 4 wks HFD + 4 wks ND n = 12, 16 wks ND n = 24, 4 wks HFD + 12 wks ND n = 12, 12 wks HFD + 4 wks ND n = 7, 24 wks ND n = 30, 12 wks HFD + 12 wks ND n = 12, 24 wks HFD n = 10, 28 wks ND n = 12, 24 wks HFD + 4 wks ND n = 8; Kruskal-Wallis test, Mann-Whitney test; **** p < 0.0001; ** p < 0.01; * p < 0.05.

General health parameters and basic reflexes were observed following the SHIRPA protocol (Fig 2). Long-time HFD caused impairment of the coat compared to age-matched control animals, which manifested in weak and scrubby fur with bare spots (Fig 2A; Kruskal-Wallis test, p = 0.0124; S1 Table, l. 18). In contrast, HFD shorter than 24 wks did not lead to obvious differences (Fig 2A). Interestingly, already a change of diet from 12 wks HFD to 4- or 12-wks ND rescued this effect (Fig 2A). Similarly, only mice receiving HFD for 24 wks showed significantly less vibrissae compared to control mice (Fig 2B; Kruskal-Wallis test, p < 0.0001; S1 Table, l. 19). Further, a longer change to ND after mid-term HFD led to preservation of the vibrissae, while a short change of diet after long-term HFD was not sufficient (Fig 2B; Mann-Whitney test, p = 0.0036; S1 Table, l. 20). No differences were observed concerning the general activity (Fig 2C) or the touch escape reactivity (Fig 2D). Further, no obvious differences exist according to the appearance of the eyes, skin, tail and gait or fecal pellets after HFD or dietary change compared to ND (S1A–S1E Fig). In addition, young and old mice as well as animals on HFD and ND showed a similar sensory reactivity testing for the vibrissae (Fig 2E), of the pinna (Fig 2F) and a moderate motor reactivity indicated by the writhe reflex (S1F Fig).

### 3.3 HFD has no effect on short-term memory

**Fig 3:**
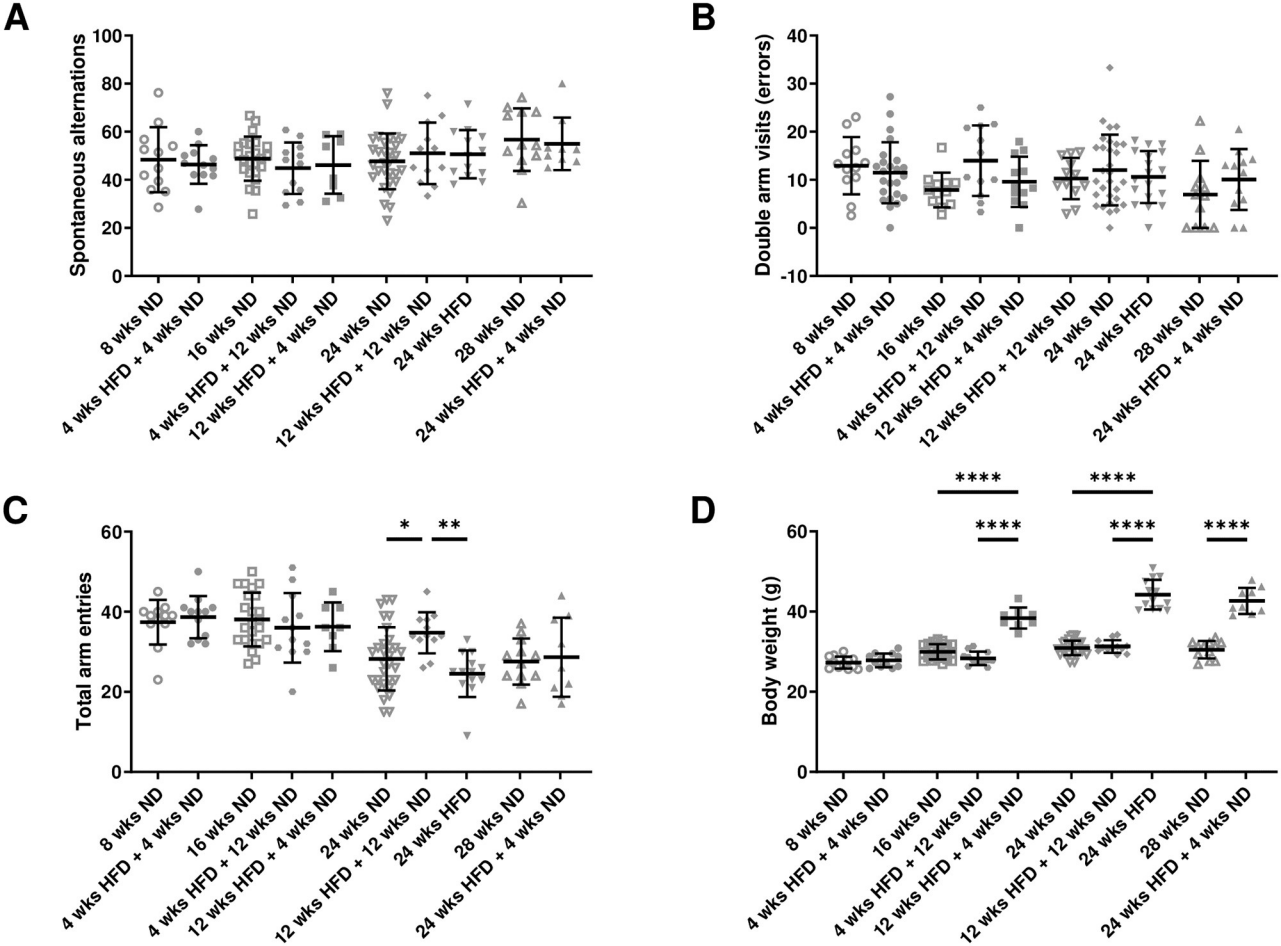
HFD has no effect on short-term memory in the Y maze test. (A) Spontaneous alternations were not affected by diet/dietary change. (B) No difference in the number of double arm visits (errors) regarding diet/dietary change was found. (C) Analysis of total arm entries revealed a significant effect of diet. (D) Mice gained more weight after longer HFD or rather lose weight after dietary change from HFD to ND. Data are presented as mean values and error bars indicate SD; 8 wks ND n = 11, 16 wks ND n = 24, 24 wks ND n = 30, 28 wks ND n = 12, 4 wks HFD + 4 wks ND n = 12, 12 wks HFD + 4 wks ND n = 8, 4 wks HFD + 12 wks ND n = 12, 12 wks HFD + 12 wks ND n = 12, 24 wks HFD n = 13, 24 wks HFD + 4 wks ND n = 9; (C) One-way ANOVA, (D) One-way ANOVA, Welch-ANOVA, unpaired t test; **** p < 0.0001; ** p < 0.01; * p < 0.05.

A potential effect of diet and dietary change on short-term memory was studied applying the Y maze test. Number of spontaneous alternations neither differed significantly between diets or after dietary change nor between young and old mice (Fig 3A). Further, we found no difference in the number of double arm visits (errors) regarding diet/dietary change or age (Fig 3B). Analysis revealed a significant effect of diet/dietary change on activity between mice being 12 wks on HFD followed by 12 wks of ND compared to age-matched mice on ND and HFD (Fig 3C; One-way ANOVA, p = 0.0021; S1 Table, l. 22). Further, there was a significant effect of age in both control and HFD groups on general activity, indicated by a decreasing number of total arm entries in the maze (Kruskal-Wallis test, p < 0.0001; One-way ANOVA, p = 0.0129; S1 Table, ll. 23–24). There was a strong effect of HFD as well as dietary change and age on body weight, though a short period of ND after long-term HFD did not rescue the phenotype (Fig 3D). Therefore, we conclude that there is no effect of long-term HFD on working memory.

### 3.4 Learning is not affected by diet

**Fig 4:**
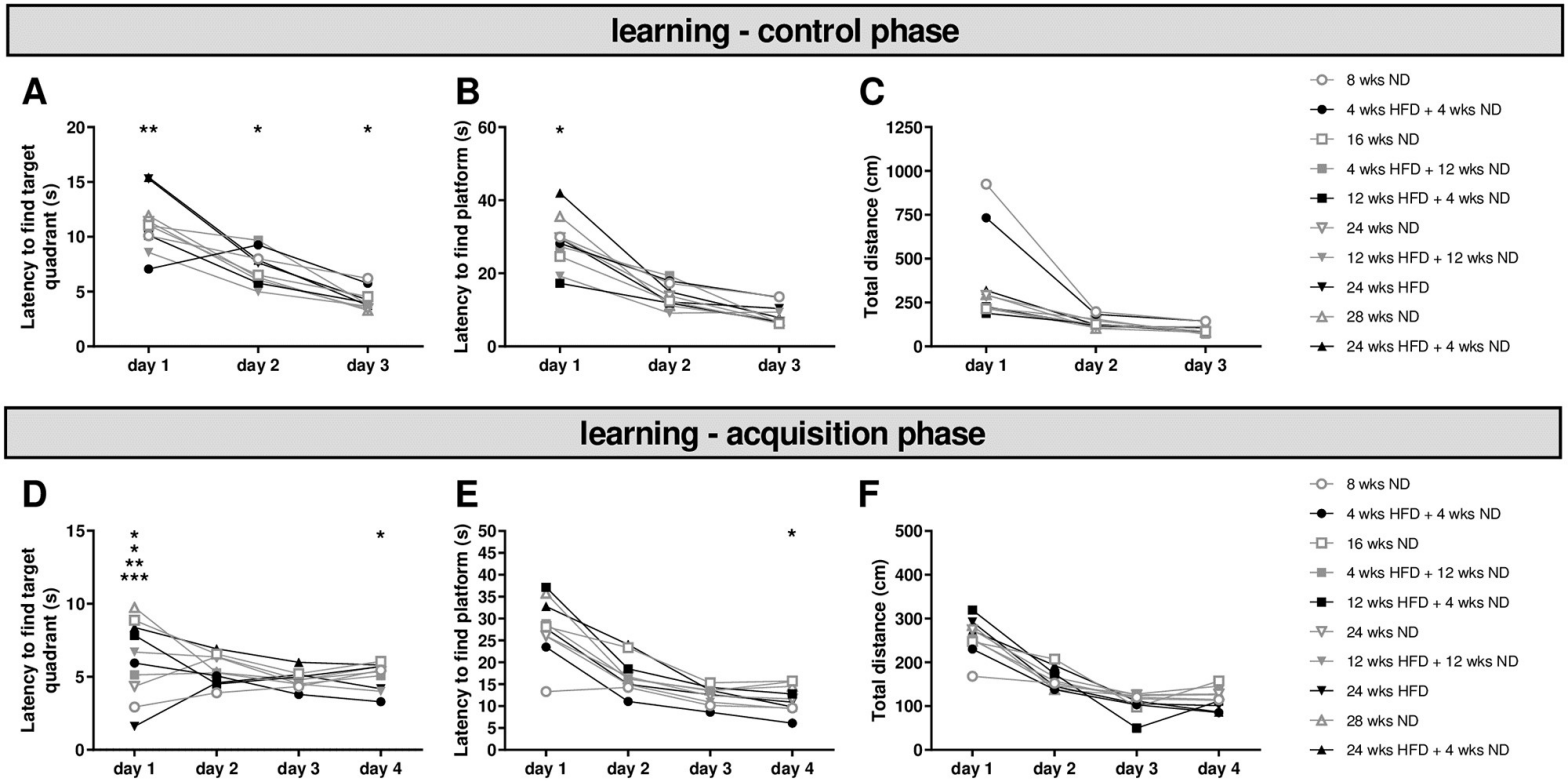
Learning is not affected by HFD. Before performing the MWM test, mice had three days to find the marked platform underneath the water by using visual information (control phase). (A) Diet had an effect on the latency to find the target quadrant. (B) We observed an effect on the latency to find the platform only on the first day regarding diet. (C) The motor activity was not affected by diet and general learning performance did not differ with respect to diet/dietary change. After the control phase, mice were trained for four days to perform the MWM test (acquisition phase). (D) Latency to find the target quadrant differed for some groups on day one and four, but learning did not change due to diet. (E) Latency to find the platform underneath the water surface showed no effect of diet. Performance up to day four did not differ relative to diet. (F) Motor activity was similar for mice on different diets. Data are presented as mean values and error bars indicate SD; 8 wks ND n = 12, 16 wks ND n = 24, 24 wks ND n = 30, 28 wks ND n = 12, 4 wks HFD + 4 wks ND n = 12, 12 wks HFD + 4 wks ND n = 7, 4 wks HFD + 12 wks ND n = 12, 12 wks HFD + 12 wks ND n = 12, 24 wks HFD n = 11, 24 wks HFD + 4 wks ND n = 9; Two-way ANOVA; *** p < 0.001; ** p < 0.01; * p < 0.05.

Analyzing the control phase of the MWM test revealed no main effect of diet or dietary change or age on learning performance (Fig 4A). Latencies to find the target quadrant were significantly higher in mice on long-term HFD compared to age-matched mice on mid-term HFD followed by a return to ND on day one and two as well as on day three in comparison to age-matched control mice (Fig 4A; Two-way ANOVA, day p < 0.0001, diet p = 0.0003, interaction p = 0.0432; S1 Table, l. 34). However, mice fed with ND for 24 wks took more time to find the target quadrant than mice being 12 wks on HFD followed by 12 wks of ND. On all days, decreased latencies to find target quadrant and platform were observed for mice receiving 12 wks ND after 12 wks HFD compared to age-matched mice on ND or HFD (Fig 4A and 4B). Almost all groups showed an improvement in learning performance regarding the latency to find target quadrant and platform (Fig 4A and 4B; S1 Table, ll. 31–63). We found no effect of diet or dietary change on activity, but an impact of age on total distance in ND and HFD groups (Fig 4C; S1 Table, ll. 64–81). Therefore, we assumed similar preconditions of age-matched mice regarding vision, motor function and learning for lean and adipose mice.

The training phase was used to estimate the learning performance after different diets or dietary change. The significant main effect of diet/dietary change according to the latency to find the target quadrant counted for day one only (Fig 4D; S1 Table, ll. 82–90). Similarly, younger animals initially showed a shorter latency to find the target quadrant (S1 Table, l. 91). Further, there was no main effect of diet on the latency to find the platform and an improvement in learning performance was observed in all groups with the exception of the study’s youngest group on ND showing consistently short latencies to find the platform from day one (Fig 4E; S1 Table, ll. 94–111). Again, we observed a significant effect of age within ND and HFD groups on the first day (S1 Table, ll. 106, 109). However, the learning performance did not differ between groups (S1 Table, ll. 107, 110). Additionally, we found no main effect of diet/dietary change on total distance and all groups with the exception of the study’s youngest group on ND increased similarly until day four (Fig 4F; S1 Table, ll. 112–123). The main effect of age on the total distance was limited to the first and third day within HFD groups and did not differ until the end of training (S1 Table, ll. 124–129). In line with the control phase, the training phase showed no differences of diet/dietary change or age on overall learning performance.

### 3.5 HFD-induced obesity does not lead to long-term memory deficits, but may alter cognitive flexibility

**Fig 5:**
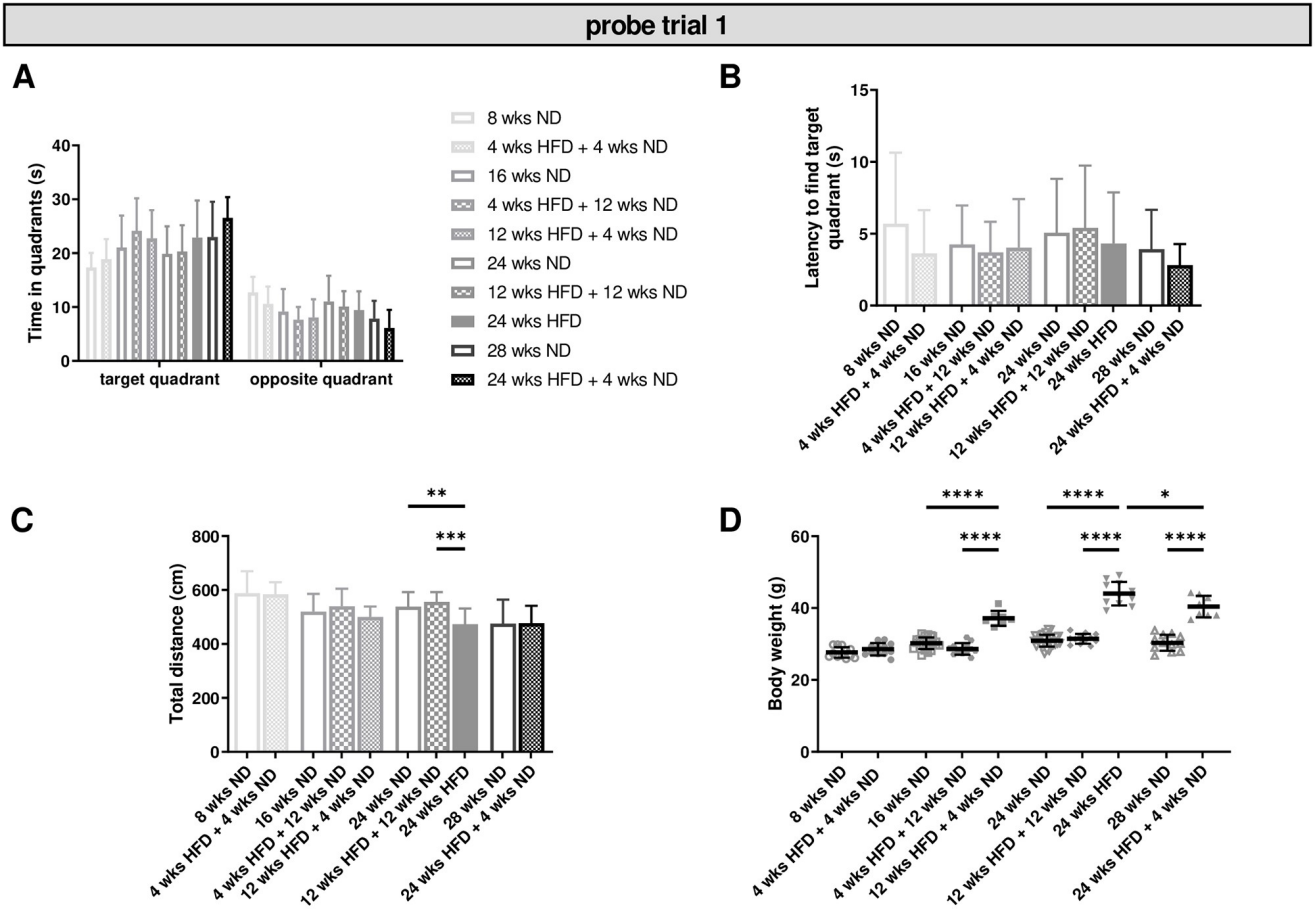
Diet-induced obesity causes no impairment of memory. Memory retention (probe trial one) was tested after four days of training in the MWM test. (A) Diet/dietary change had no effect on the time mice spent in quadrants, while older animals spent more time in the target quadrant. (B) No effect was found regarding the latency to find the target quadrant. (C) Motor activities were reduced in mice receiving 24 wks of HFD compared to age-matched control mice. (D) Diet had a significant effect on body weight and mice benefited from a change to ND after HFD. Data are presented as mean values and error bars indicate SD; 8 wks ND n = 12, 16 wks ND n = 24, 24 wks ND n = 30, 28 wks ND n = 12, 4 wks HFD + 4 wks ND n = 12, 12 wks HFD + 4 wks ND n = 7, 4 wks HFD + 12 wks ND n = 12, 12 wks HFD + 12 wks ND n = 12, 24 wks HFD n = 11, 24 wks HFD + 4 wks ND n = 8; (A): Two-way ANOVA, (B, C, D): One-way ANOVA, Welch-ANOVA, unpaired t test; ****; p < 0.0001; *** p < 0.001; ** p < 0.01; * p < 0.05.

In probe trial one, we observed no effect of diet/dietary change on time spent in the target quadrant and all groups of mice showed a significant discrimination between the target and the opposite quadrant (Fig 5A; S1 Table, ll. 130–141). In contrast, mice with increasing age spent more time in the target and respectively less time in the opposite quadrant within HFD groups (S1 Table, ll. 142–147). Further, there was no effect of diet/dietary change or age on the latency to find the target quadrant (Fig 5B). Total distance decreased in mice on long-term HFD compared to age-matched control groups (Fig 5C; One-way ANOVA, p = 0.0006; S1 Table, l. 148). The motor activity also decreased with age within ND and HFD groups (S1 Table, ll. 149–150). As expected, mice receiving HFD gained significant more weight than mice on ND and animals benefited significantly from dietary change (Fig 5D; S1 Table, ll. 151–154). Body weight enhanced also with increasing age independent of diet (S1 Table, ll. 155–157). No effect of diet/dietary change or age was found relative to the latency to find the platform location. In sum, we found no effect of diet/dietary change on test performance, but observed a negative effect of age within ND and HFD groups. However, our data revealed a strong effect of diet/dietary change and age on body weight. Further, long-term HFD and advanced age also resulted in decreased motor activity.

**Fig 6:**
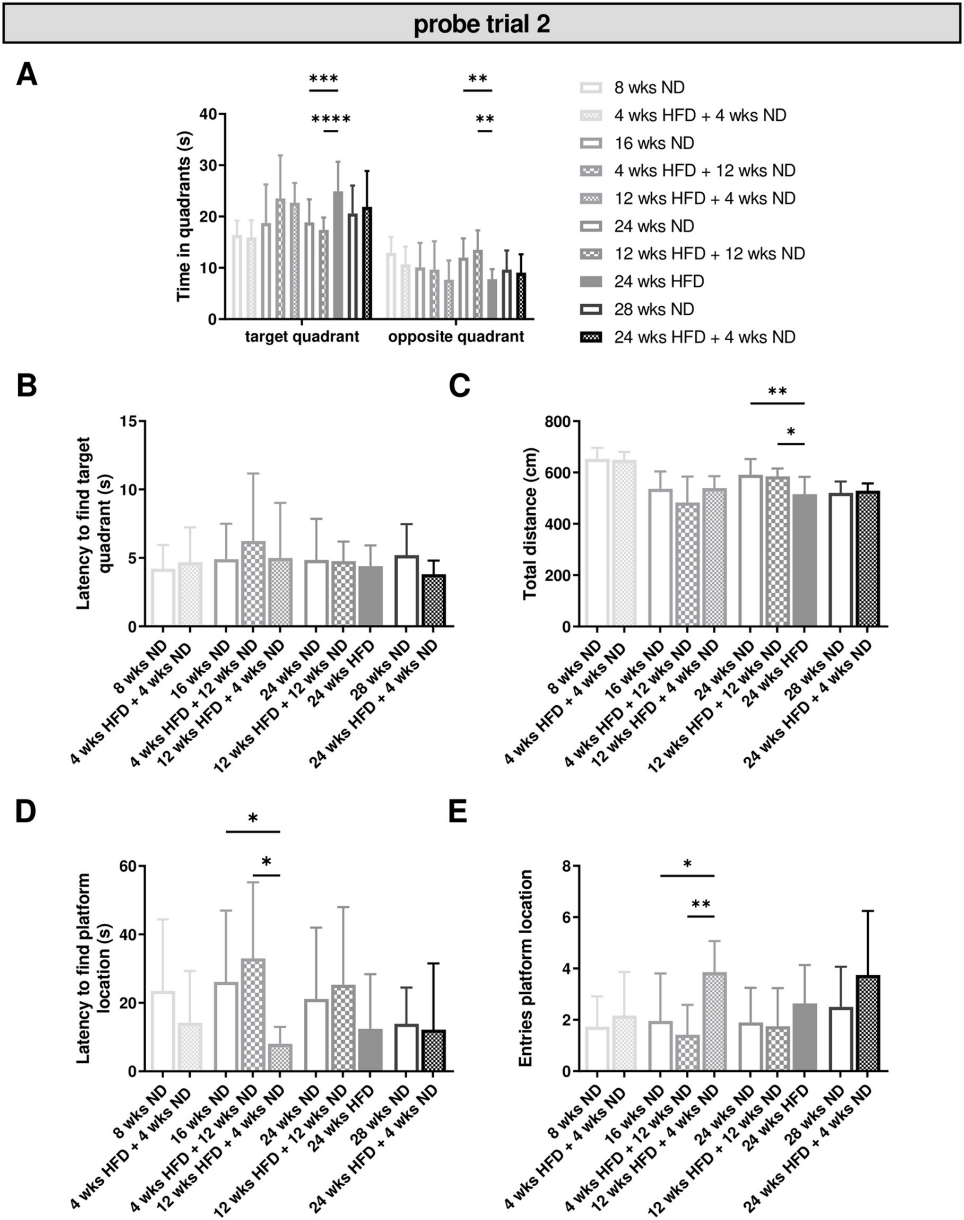
HFD leads to intact long-term memory, but may impair cognitive flexibility. Memory retention (probe trial two) was tested again after two days without any training in the MWM test. (A) Time mice spent in the target quadrant was significantly increased in mice receiving 24 wks of HFD compared to age-matched control mice. (B) No effect of diet was observed relative to the latency to find the target quadrant. (C) Diet had an effect on motor activity. (D, E) Dietary change showed a shorter latency to find the platform location and a significant effect on the entries of the platform location. Data are presented as mean values and error bars indicate SD; 8 wks ND n = 12, 16 wks ND n = 12, 24 wks ND n = 18, 28 wks ND n = 12, 4 wks HFD + 4 wks ND n = 12, 12 wks HFD + 4 wks ND n = 7, 4 wks HFD + 12 wks ND n = 12, 12 wks HFD + 12 wks ND n = 12, 24 wks HFD n = 10, 24 wks HFD + 4 wks ND n = 8; (A): Two-way ANOVA, (C): One-way ANOVA, (D, E): Kruskal-Wallis test; **** p < 0.0001; *** p < 0.001; ** p < 0.01; * p < 0.05.

In a second probe trial, we observed no overall effect of diet/dietary change on time spent in the target or opposite quadrant. However, mice fed with HFD for 24 wks stayed significantly longer in the target quadrant compared to age-matched control groups (Fig 6A; two-way ANOVA, quadrant p < 0.0001, diet p = 0.3411, interaction p < 0.0001; S1 Table, ll. 164–166). Just as in probe trial one, we observed no effect of diet and age relative to the latency to find the target quadrant (Fig 6B). The motor activity was significantly reduced after long-term HFD of 24 wks compared to age-matched control animals (Fig 6C; One-way ANOVA, p = 0.0016; S1 Table, l. 173) and showed a significant effect of age within ND and HFD groups (S1 Table, ll. 174–175). Further, we observed a reduced latency and significant more entries of the platform location in mice with a short ND time after 12 wks of HFD compared to four wks of HFD followed by a longer ND period, while there was no effect of age (Fig 6D and 6E; Kruskal-Wallis test, latency p = 0.0164, entries p = 0.0052; S1 Table, ll. 176–177).

In sum, our investigations revealed no effect of diet/dietary change, but of age on memory performance with a strong effect of age and diet/dietary change on body weight. While we observed no effect of diet or age relative to the platform location during the first probe trial, there was an effect of dietary change in the probe trial two.

### 3.6 HFD has no consequences for the emotional state

In order to test whether HFD-induced obesity leads to alterations of the emotional condition (e.g. motivation), we conducted the tail suspension test. Healthy mice are meant to try to escape by active movements, when fixed and suspended by their tails. In our study no effect of diet/dietary change on activity was detected, while older mice within ND groups were more active in this test (S2A Fig; S1 Table, ll. 183–184). However, we observed no difference of diet/dietary change or between mice of different age according to the latency to inactivity (S2B Fig). Therefore, we conclude that there are no consequences of HFD on the emotional state in mice.

### 3.7 Increased microglial activation after long periods of HFD in the hypothalamus

**Fig 7:**
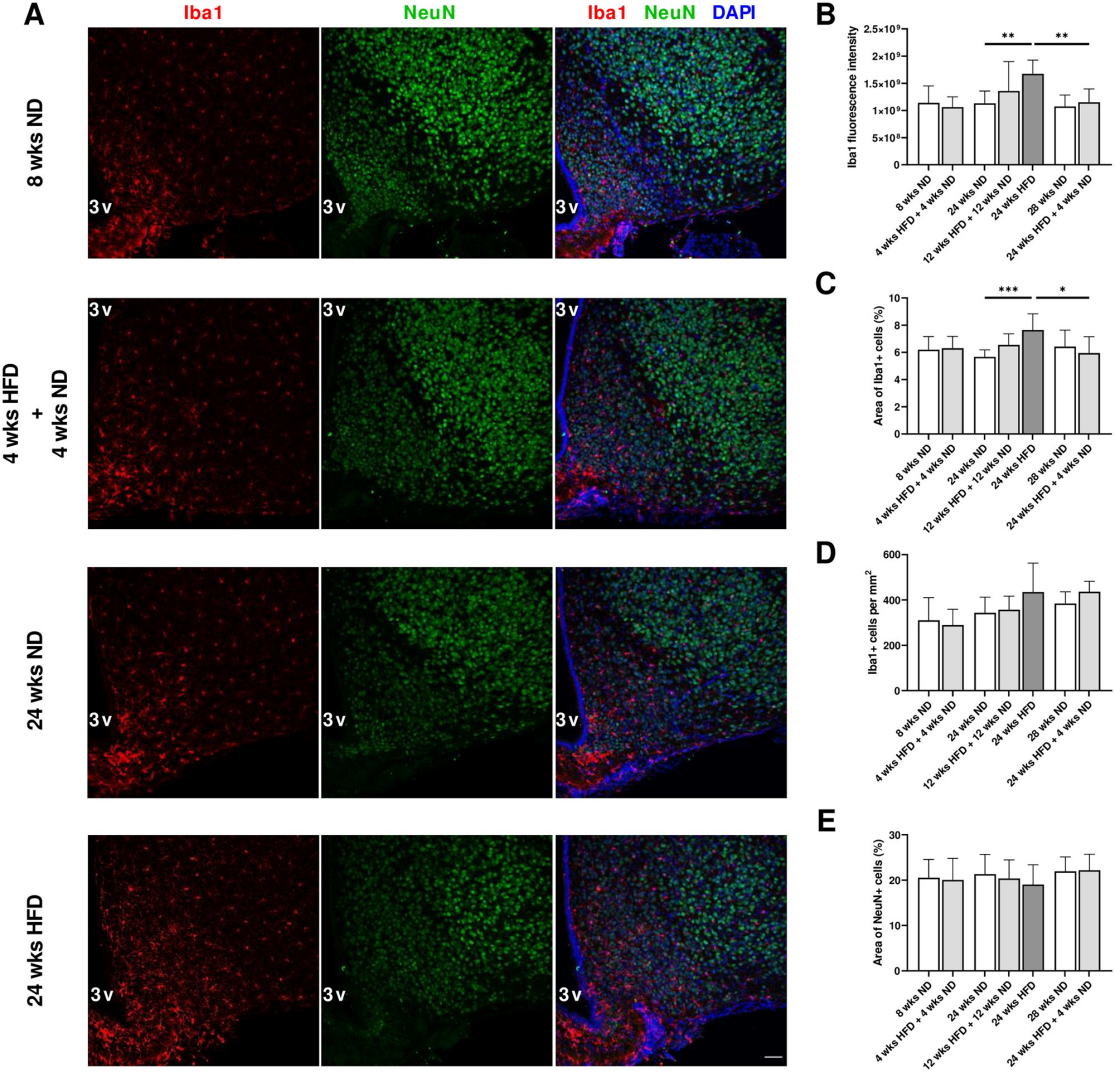
Increased microglial activation after long periods of HFD in the hypothalamus. (A) Representative confocal images of double-labeled immunofluorescence staining for Iba1 (red) and NeuN (green) within the mediobasal hypothalamus of male wild-type C57BL/6J mice fed with ND for eight wks, HFD for four wks followed by ND for four wks and ND or rather HFD for 24 wks. Nuclei were counterstained with DAPI. Scale bar corresponds to 50 μm; 3v, third ventricle. (B) Fluorescence intensity measurements of Iba1, (C) quantification of percentage of area covered by Iba1 and (E) NeuN-immunoreactive profiles and (D) number of Iba1-immunoreactive cells revealed a microgliosis in response to long-term HFD exposure without effects on neurons. Data are presented as mean values and error bars indicate SD; 8 wks ND n = 6, 4 wks HFD + 4 wks ND n = 6, 24 wks ND n = 12, 12 wks HFD + 12 wks ND n = 6, 24 wks HFD n = 6, 28 wks ND n = 6, 24 wks HFD + 4 wks ND n = 6; (B): Welch-ANOVA, unpaired t test, (C): One-way ANOVA, unpaired t test; *** p < 0.001; ** p < 0.01; * p < 0.05.

We performed immunostaining on coronal brain sections of the youngest mice (8 wks ND vs. 4 wks HFD + 4 wks ND), animals that received mid-term HFD followed by a dietary change back to ND for the same period of time (24 wks ND vs. 12 wks HFD + 12 wks ND), older mice on long-term HFD (24 wks ND vs. 24 wks HFD) and with dietary change (28 wks ND vs. 24 wks HFD + 4 wks ND) to assess the effect of diet/dietary change on microglial and neuronal morphology in the hypothalamus (n = 6–12 mice per condition). Microglial response determined by Iba1 signal intensity increased after 24 wks of HFD (Fig 7A and 7B; Welch-ANOVA, p = 0.0069; S1 Table, l. 178). Similarly, the area covered by Iba1-positive cells was significantly enhanced after 24 wks of HFD compared to ND for 24 wks (Fig 7C; One-way ANOVA, p = 0.0003; S1 Table, l. 180). Interestingly, long-term HFD (24 wks) followed by a dietary change back to ND resulted in lower Iba1 fluorescence intensity and smaller area of Iba1-positive cells (Fig 7B and 7C; S1 Table, ll. 179, 181). Short- and mid-term HFD (four and 12 wks) followed by a dietary change back to ND did not lead to higher fluorescence intensities and percentage of Iba1-stained area compared to age-matched control mice (Fig 7B and 7C). However, we could detect a trend towards higher fluorescence intensity and percentage of stained area with Iba1 after 12 wks on HFD followed by 12 wks on ND and 24 wks on HFD followed by four wks on ND compared to 24 wks on ND (Fig 7B and 7C). Indeed, in mice fed with ND or HFD followed by dietary change, we predominantly observed the ramified form of microglia exhibiting small somata and fine ramifications, while in the hypothalamus of mice on long-term HFD, many microglial cells displayed an activated morphology with thickened processes and bigger cell bodies. Number of Iba1-positive cells was slightly elevated following long-term HFD, but the difference between 24 wks on ND and 24 wks on HFD was not significant (Fig 7D; One-way ANOVA, p = 0.1164). Further, hypothalamic expression of mRNA encoding *Iba1* increased by 25% in mice fed a HFD for 24 wks (S3 Fig; Two-tailed unpaired t test, p = 0.0328; S1 Table, l. 185). We did not observe morphological alterations in neurons within the mediobasal hypothalamus after a dietary change or a long-term HFD (Fig 7A and 7E). In summary, long periods of HFD increased microglial activation within the mediobasal hypothalamus without effects on neuronal morphology and NeuN-stained areas as a substitute for cell numbers.

### 3.8 HFD-induced obesity does not alter cortical and hippocampal morphology

**Fig 8:**
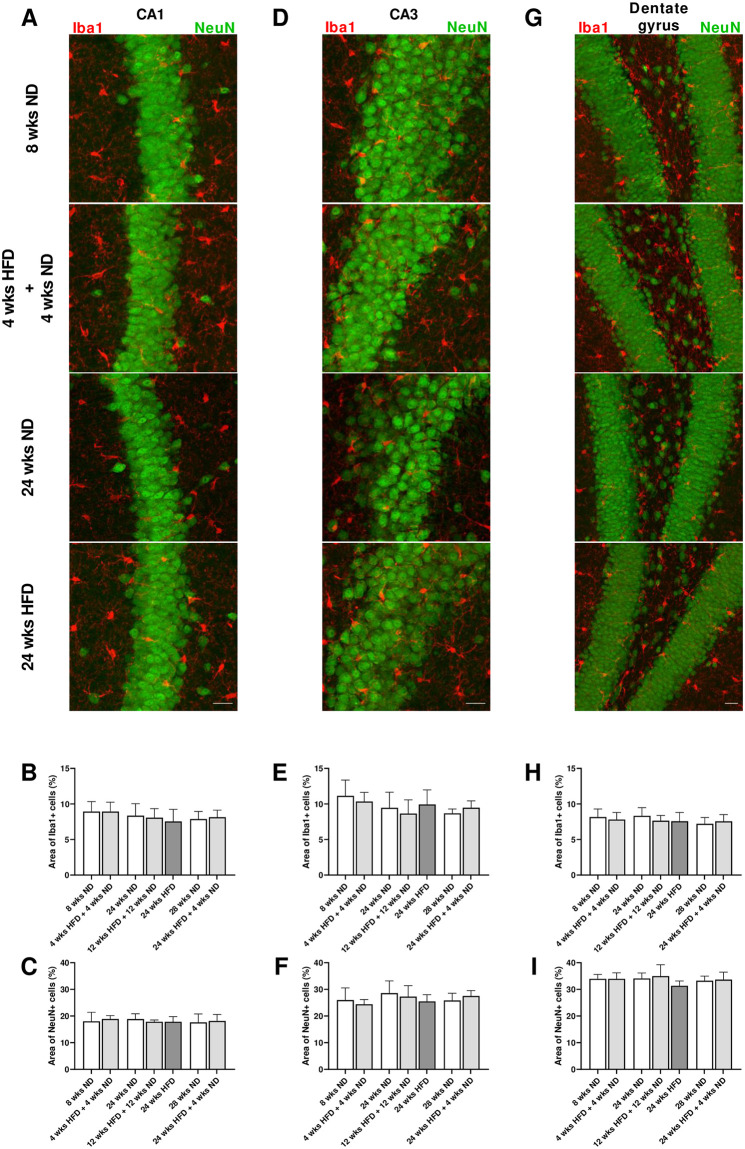
HFD-induced obesity does not alter hippocampal morphology. (A, D, G) Representative confocal images of double-labeled immunofluorescence staining for Iba1 (red) and NeuN (green) within the hippocampus, in particular CA1 and CA3 regions and dentate gyrus of male wild-type C57BL/6J mice fed with ND for eight wks, HFD for four wks followed by ND for four wks and ND or rather HFD for 24 wks. (B, E, H) Quantification of the area stained by Iba1 and (C, F, I) NeuN revealed no differences in hippocampal morphology in mice on HFD compared to ND-fed mice. Scale bar corresponds to 25 μm. Data are presented as mean values and error bars indicate SD; 8 wks ND n = 6, 4 wks HFD + 4 wks ND n = 6, 24 wks ND n = 12, 12 wks HFD + 12 wks ND n = 6, 24 wks HFD n = 6, 28 wks ND n = 6, 24 wks HFD + 4 wks ND n = 6.

There were no differences regarding fluorescence intensity and percentage of area of both Iba1 and NeuN immunosignals between the analyzed groups within the frontal motor cortex (S4A–S4C and S4E Fig). Number of Iba1-postive cells was not altered after diet/dietary change compared to age-matched mice (S4D Fig). Similarly, we found no alterations within hippocampal areas CA1, CA3 and dentate gyrus after HFD exposure and dietary change compared to age-matched control groups (Fig 8A–8I). Microglial cell density within hippocampal areas did not change neither after diet/dietary change nor with age (S5 Fig).

A more detailed quantitative analysis of microglial morphology in hippocampal brain regions revealed that in CA1, microglial cell area, perimeter, convex hull area and skeleton length were significantly elevated in animals that received mid-term HFD followed by a dietary change back to ND for the same time period compared to age-matched mice (S6A–S6C and S6F Fig; One-way ANOVA, A) p = 0.0240, B) p = 0.0305, C) p = 0.0226, F) p = 0.0349; S1 Table, ll. 186–189). On the other hand, averaged microglial cell solidity and soma size did not differ significantly between relevant groups in CA1 (S6D and S6E Fig). CA3 and hilar regions did not show significant alterations in morphological parameters of microglial cells after diet/dietary change (S6G–S6R Fig). However, microglial cell area, perimeter, convex hull area and skeleton length were also slightly elevated in animals that received mid-term HFD followed by a dietary change back to ND for the same time period compared to age-matched mice (S6G–S6I, S6L–S6O and S6R Fig). Microglial cells in these mice had slightly bigger cells of the ramified morphology showing similar cell solidities and soma size areas, but also longer skeleton lengths and higher branching indices compared to age-matched control mice leading to bigger cell area, perimeter and convex hull area (S6G–S6R Fig; S2 Table). In general, ramified microglial cells have smaller soma areas, bigger cell perimeters and longer skeleton lengths than activated microglia. Microglial cell and soma area are expected to increase due to activation and soma enlargement yielding higher values of these morphological parameters for activated microglia. Soma size area was not higher in the group that received mid-term HFD followed by a dietary change back to ND for the same time period (S6K and S6Q Fig). Microglia of these mice had larger cell perimeters, which is estimated to be higher in ramified cells and its decrease is characteristic of fewer ramifications (S6L and S6R Fig). Increasingly ramified cells have larger convex hull areas and lower cell solidity. An increase of this parameter, also known as cell occupancy, reveals the tendency of microglial cells to be more compact indicating the transition toward activation. Cell circularity is expected to be higher for activated microglia. Typically, highly ramified microglial cells have a greater skeleton length, as well as many branch and end points. The branching index is an additional measurement of microglial branching complexity. For instance, a small ramified microglial cell and an activated microglial cell may have a similar cell volume, but the activated state occupies more of its surrounding, therefore the branching index measure will be smaller. Various important morphological parameters (or their appropriate combination) which indicate microglial activation did not differ in hippocampal regions between relevant groups [60]. Quantification of microglial morphological parameters revealed the most significant changes in the frontal cortex with age, but not with diet/dietary change (S6S–S6X Fig). Within cortical regions, microglia of older mice showed higher values for cell area (S1 Table, ll. 190–191; S2 Table), cell perimeter (S1 Table, ll. 192–193; S2 Table), convex hull area (S1 Table, ll. 194–195; S2 Table) and skeleton length (S1 Table, ll. 196–197; S2 Table). There were no detectable changes in relevant morphological parameters indicating microglial activation neither with age nor with diet/dietary change.

Taken together, we conclude that brains of mice on ND and HFD have similar structural features within the frontal motor cortex and the hippocampus. Quantitative analysis of morphological parameters of microglial cells reveals no microglial activation within the hippocampus and cortex after long-term HFD.

### 3.9 Long-term HFD does not lead to lipid droplet accumulation and histological changes in the hippocampus

**Fig 9:**
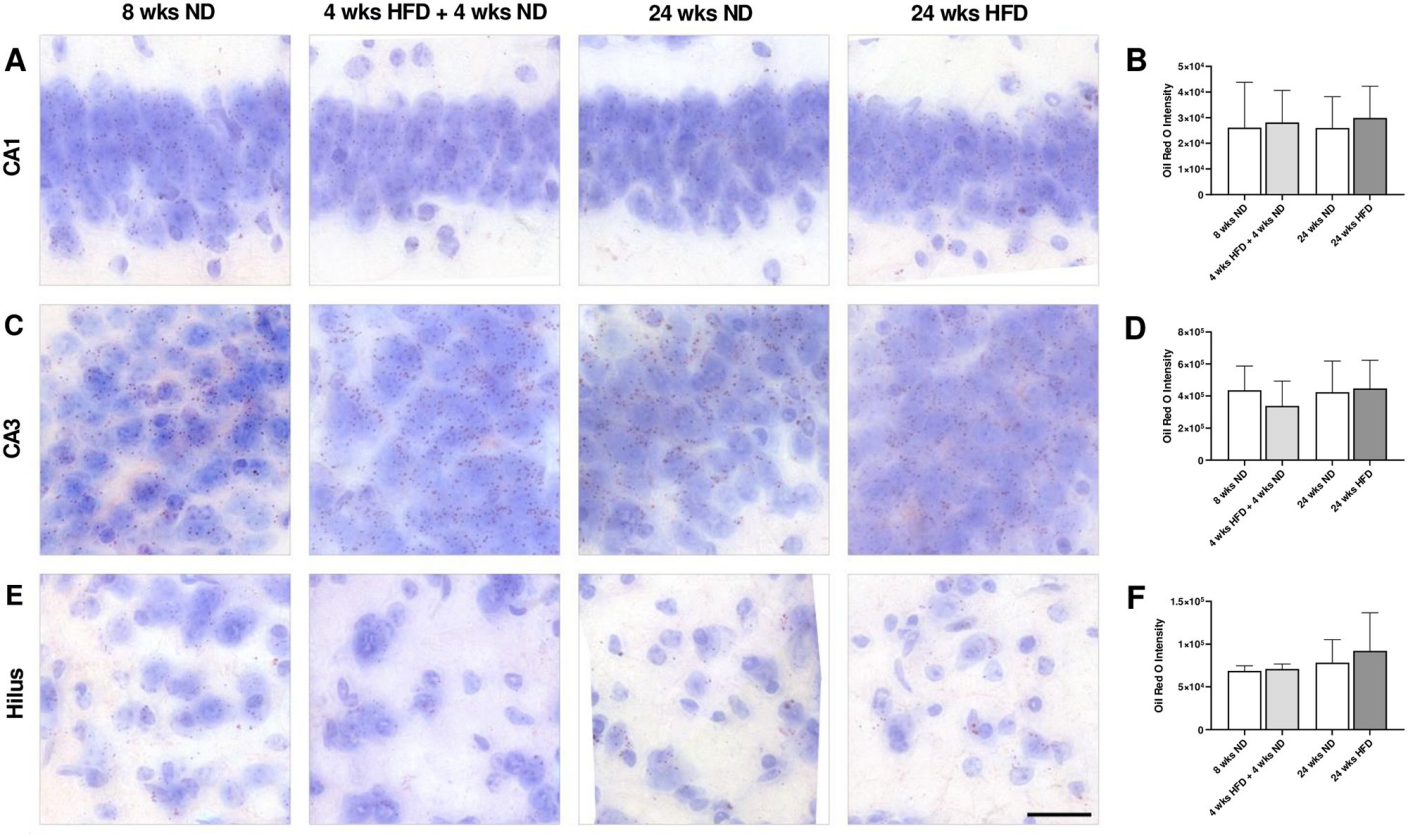
Long-term HFD does not lead to lipid droplet accumulation and histological changes in the hippocampus. (A, C, E) Representative photomicrographs showing Oil Red O stained CA1, CA3 and hilar regions of male wild-type C57BL/6J mice fed with ND for eight wks, HFD for four wks followed by ND for four wks, ND or rather HFD for 24 wks. (E) Scale bar corresponds to 25 μm. Graphs display (B) Oil Red O intensity in CA1, (D) CA3 and (F) hilar regions. Dietary lipids do not accumulate within the hippocampus due to HFD. Data are presented as mean values and error bars indicate SD; 8 wks ND n = 4–5, 4 wks HFD + 4 wks ND n = 5–6, 24 wks ND n = 6, 24 wks HFD n = 6.

In order to find out whether dietary lipids reach the brain and accumulate there over time, we analyzed coronal brain sections stained with Oil Red O of the study’s youngest group 8 wks ND vs. 4 wks HFD + 4 wks ND and the oldest group 24 wks ND vs. 24 wks HFD undergoing the longest period of HFD without dietary change. Oil Red O-stained droplets were predominantly distributed in the hippocampus, in particular in CA1, CA3 and hilar regions. However, lipid droplet accumulation did not increase in CA1 and CA3 neurons and hilar areas in HFD-fed mice compared to ND-fed mice (Fig 9A, 9C and 9E). Further, Oil Red O intensity of stained lipid droplets did not differ between ND- and HFD-fed mice (Fig 9B, 9D and 9F). Calculated ratios (lipid droplets per cell, per tissue, per cell area, and per tissue area) did not reveal any differences in lipid droplet accumulation neither after a long period of HFD nor with age (S7 Fig). Furthermore, ND- and HFD-fed mice had a normal histological appearance and neuronal distribution in CA1 and CA3 hippocampal regions (Fig 9A, 9C and 9E). Overall, results of Oil Red O staining suggested that long-term HFD does not cause structural damage in CA1 and CA3 neuronal cells.

## 4. Discussion

### 4.1 Long periods of HFD induce microglial activation in the hypothalamus, but not in the hippocampus

Previous studies already reported about inflammatory microglial responses to variable durations of HFD in the hypothalamus, where microglia, the CNS analogs of macrophages, and astrocytes are involved in body weight homeostasis and obesity [37, 45, 65, 66]. However, hypothalamic inflammation and microgliosis, characterized by rapid morphological changes and microglial inflammatory signals, have been found to occur very early in response to a fat-dense diet [45, 66–69]. A study by Waise et al. (2015) showed that even one day of HFD induced inflammation in the hypothalamus exhibiting increased numbers of macrophages/microglia and upregulated mRNA expression levels of proinflammatory biomarkers [69]. Further, it was recently demonstrated that microglia, bone-marrow derived macrophages, may maintain their pool without significant substitution by circulating monocytes after their establishment in early postnatal life, even under conditions of chronic HFD feeding [70]. Here, for immunofluorescence labeling of microglial cells in hypothalamic sections, we used Iba1, which is a molecular marker for active microglia participating in membrane ruffling and phagocytosis [59]. Iba1 is also known to increase the expression of inflammatory chemokines and cytokines [71]. In our study, we found that long periods of HFD lead to microglial activation within the mediobasal hypothalamus. Interestingly, a dietary change after four, 12 and 24 wks of HFD back to ND rescued this effect already observed in the aforementioned HFD feeding studies for short- and mid-term HFD exposure (Valdearcos et al., 2014, 2017; Baufeld et al., 2016). Berkseth and colleagues (2014) previously reported that hypothalamic gliosis associated with 16 wks HFD exposure is largely reversible in rodents, consistent with reversal of the HFD-induced obesity phenotype [72]. This study also demonstrated that POMC neuronal cell number did not differ between ND, HFD, HFD/ND groups. Here, the activation of microglial cells after long-term HFD did not coincide with morphological alterations or signs of degeneration of neurons within the mediobasal hypothalamus. Unfortunately, the morphological analysis failed within the hypothalamus due to many overlapping cells and inadequate detection of cell bodies. However, this brain area was not the focus of our study and our results from Iba1 signal intensity and stained area quantification after HFD exposure are largely confirmatory of published studies [37, 45]. In contrast to our findings in the hypothalamus responsible for appetite and weight control, we did not discover morphological changes in microglia and neurons of the hippocampus, a brain region required for memory and learning, after long-lasting HFD exposure. In line, Agrimi et al. (2019) showed that 18 wks of HFD did not modify the morphology of the hippocampal formation in adult mice [73]. However, it was previously demonstrated that consumption of HFD during the juvenile period impaired hippocampal morphology and function, as reviewed by Del Olmo and Ruiz-Gayo (2018) [74]. Also, early exposure to HFD induces inflammatory changes in the mouse hippocampus [75]. In this study, six wks of HFD in three-wks-old C57BL/6J male mice increased Iba1-positive cells’ soma area in the hilus and stratum radiatum when comparing to ND. Here, we focused on CA1, CA3 and dentate gyrus/hilar regions, where no microglial activation after long periods of HFD was observed. Hence, the starting age and duration of HFD need to be considered regarding the evaluation and comparison of microglial responses in specific brain regions.

### 4.2 HFD-induced obesity has no effects on hippocampal morphology and lipid droplet contents

Adequate nutritional status and dietary intake are essential for healthy brain functioning. Emotions, behavior, cognitive processes, neuroendocrine functions and synaptic plasticity can be affected by excessive nutritional intake with possible detrimental outcome on neuronal physiology. Lipids, in particular, have important effects on neuronal structure and function [76]. It is known that in metabolic diseases and during aging lipid droplets, which are cytoplasmic non-polar lipids with lipophilic constitutive proteins on their surface, accumulate in many organs including the liver, muscle and brain. Lipid droplets were identified in meningeal, cortical and neurogenic brain regions corresponding to distinct cellular phenotypes, including astrocytes, microglial and neuronal cells, during aging by Shimabukuro et al. (2016) [77]. In our study, we assessed the consequences of HFD-induced obesity on lipid droplet content in the brain using Oil Red O staining. Lipid droplets were predominantly distributed in the hippocampus in ND- and HFD-fed mice, but there was no evidence of an increased lipid droplet accumulation in CA1, CA3 and hilar regions after long-lasting HFD exposure. Thus, our results indicate that the lipid droplet contents within the hippocampus do not increase by long-term HFD and lipid droplets do not accumulate in hippocampal regions and neurons with age. In contrast to our findings, Zhao et al. (2017) described a slight increase in lipid contents by Oil Red O staining in CA3 of the hippocampus in apolipoprotein E-deficient (ApoE -/-) mice after receiving HFD for 12 wks [78]. Unfortunately, quantitative analysis between ND and HFD is missing in this study. Further, they observed neuronal loss in the CA3 provoked by hyperlipidemia. In line, CA1 region was not affected by mid-term HFD exposure. The disagreements regarding the CA3 region are probably caused by the usage of the ApoE knockout mouse line, a suitable model to study hyperlipidemia, which is a risk factor for neurodegenerative diseases, including Alzheimer’s disease. ApoE, the most abundant apolipoprotein in the CNS, is particularly implicated in cholesterol efflux and microglial phagocytosis. A review by Loving and Bruce (2020) suggested that increased ApoE production in microglia is a response to increased intracellular cholesterol accumulation [79]. The diet composition is another important aspect that needs to be considered when comparing feeding studies in general. In the study of Zhao et al. (2017), diet consisted of 10% lard, 2% cholesterol and 0.5% cholic acid [78], whereas our experimental diet, characterized by extremely high amounts of fat with middle-chain saturated fatty acids (coconut oil), contained 20.3% crude protein, 35.5% crude fat, 0.1% crude fiber, 5.3% crude ash, 0.2% starch, 17.0% sugar and no additional cholesterol. Maya-Monteiro and colleagues recently showed that lipid droplets accumulate in the hypothalamic third ventricle wall layer with similar heterogeneous distributions in human and mouse [80]. The HFD used in this study also contained high carbohydrates (17 kcal% protein, 42 kcal% carbohydrate, and 41 kcal% fat as well as a high-fructose corn syrup sweetened beverage), whereas our experimental diet consisted of less carbohydrates and more fat (59 kcal% fat, 26 kcal% carbohydrates, 15 kcal% protein). In contrast to our lipid staining, the authors used immunofluorescence labeling of the specific lipid droplet protein PLIN2 to visualize and quantify lipid droplets.

Moreover, in addition to histological analysis, we aimed to study effects after long-term HFD (24 wks) on general health, emotional state, learning and memory in mice. Further, we wanted to analyze potential improvement of health and behavioral performance after the diet has been changed back to a ND, which has hardly been described in the literature so far [72, 81].

### 4.3 Long-term HFD and obesity lead to health issues, without affecting reflexes or the emotional state in mice

It is known that a fat-dense diet and obesity can cause impairments of general health such as metabolic alterations and cardiac dysfunction [73, 82–84]. Using the SHIRPA protocol and body weight we assessed the consequences of long-term HFD and obesity, as well as dietary change, on appearance in mice. Here, we found that applying a fat-dense diet for mid- and long-term increased the body weight significantly. Further, a change of diet back to ND, after having HFD before, revealed a positive effect on the body weight and hepatic lipid accumulation. Long-term HFD led also to weak and scrubby fur and bare spots, which is an indication for health problems in animals. Interestingly, already a short dietary change to ND helped to avoid this consequence. Similarly, mice on long-term fat-dense diet showed significant more vibrissae loss, while already 12 wks of ND were sufficient for preservation of the whiskers. Loss of vibrissae is often a result of conflicts within the group of mice housed together [85–87]. Thus, long-term HFD may facilitate antisocial behavior and increase aggression, which can also affect general health later on. SHIRPA analysis indicated no differences in aggression toward the experimenter, while social behavior was not studied here. Future studies could apply the intruder paradigm with animals of the same group to investigate whether HFD increases aggression. Interestingly, we observed no further differences between mice on HFD compared to ND relative to eyes, skin, tail, gait, general activity or the touch escape. Also, sensory and motor reflexes were without any pathological findings, which is important to evaluate results from other behavioral tests. As an association between obesity and depression had already been described [88–90], we asked whether obese mice following HFD develop depressive-like features. A study by Vagena et al. (2019) with C57BL/6J mice showed that already three wks of fat-dense diet cause depressive-like behavior in the tail suspension and the forced swim test [91]. In order to test whether depressive-like behavior influences the motivation in cognitive and memory tests, we applied the tail suspension test as well. However, our data showed no evidence for depressive-like behavior in this test. Together, we could rule out that the motivation or performance in other behavioral tests were influenced by alterations of the emotional state, severe motor problems or constraints of sensory reflexes.

### 4.4 Diet-induced obesity has no effect on learning and memory, but may influence cognitive ability

Many studies describe a link between obesity and learning deficits, memory and cognitive impairments, as well as an increased risk for dementia and Alzheimer’s disease [83, 92, 93]. Unfortunately, most studies refer to different study designs, applied tests and parameters to evaluate a diet-induced memory impairment. To assess an effect of long-term HFD and dietary change on working memory, which depends on hippocampal effort [94], we applied the Y maze test. Animals with impaired working memory show increased number of errors and altered spontaneous alternations in this test [95, 96]. Our study revealed no effect of HFD, dietary change or age on short-term memory regarding these parameters. But, a stable effect of age on activity was found in both HFD and ND groups, showing a decreasing number of arm entries. In line, Agrimi et al. (2019) described that 18 wks of HFD alone did not impair spatial memory in the Y maze test [73]. To develop cognitive deficits other co-existing risk factors such as psychosocial stress were needed. Also, no differences in the Y maze test were found in mice, whose mothers received HFD during gestation [97].

Further, learning and memory were analyzed using the MWM test. Thereby, we conducted a control phase, where mice were trained to swim to the labeled platform underneath the water first, which is in contrast to many other studies. This phase was used to evaluate the qualification of obese mice to pass the test regarding vision, motor function and learning, compared to lean mice. In the control and in the acquisition phase, where mice were trained to find the platform underneath the water by visual cues in the environment, we observed no effect of diet or dietary change. Only initial effects were found relative to age. Similar to the control phase, performance improved over testing days and training revealed no effect of diet/dietary change or age on overall learning. Therefore, we could assure that aged-matched mice receiving ND and HFD possessed similar preconditions regarding vision, motor function and learning in the MWM test.

Directly after the acquisition phase, memory retention was tested in probe trial one in which the platform was removed. Analyzing latency and time in the target quadrant revealed no effect of diet or dietary change on test performance. As we observed also no effect of diet or age according to the latency to find the platform location, we suppose even long-term HFD does not cause memory impairment. Besides, long-lasting HFD and high age can affect the motor performance in mice. However, age-dependent effects were not the focus of our study. As before, we observed no effect of diet or dietary change on memory performance in probe trial two, performed after two days without any training. Contrary, in the second probe trial a tendency in latency and a difference in platform entries were found for mice on dietary change. Mice on longer HFD (12 wks HFD + 4 wks ND) seemed to stick to the old platform location learned during training, while age-matched mice on shorter HFD (4 wks HFD + 12 wks ND) rather ignored the previous platform location. Mice may have learned from the experience in probe trial one that the platform is not at the same position and could be located elsewhere. Further, compared to age-matched groups, mice on long-term HFD (24 wks) spent more time in the target quadrant, where the platform was supposed to be. Indeed, it could be more efficient to check other quadrants and use another searching strategy. According to this, we hypothesize that the cognitive flexibility and memory extinction may be impaired by HFD, so that these mice stick to their old searching strategy and prefer the known platform location [98–100]. In line, a study by Woo et al. (2013), measuring cognitive function-related proteins such as NGF (nerve growth factor) and BDNF (brain-derived neurotrophic factor), suggested a decrease in plasticity and cognitive function in the brain of rats maintained on HFD [101]. Further, they indicated enhanced protein and mRNA expression levels after dietary change. Supporting our data, Jurdak et al. (2008) showed that sugar-induced obesity leads to impairment in spatial learning and memory in the MWM in young rats, while fat-induced obesity is not sufficient for behavioral alterations [102].

Comparing studies of diet-induced obesity effects on learning and memory impairment showed a high discrepancy with reference to species, sex, age, analyzed parameters, applied tests, and particularly the feeding paradigm such as diet (high-fat, high-sugar or combined diets, start and duration). In terms of mouse line, a study with APP23 mice, a model for Alzheimer´s disease, 12-month-old animals fed for 12 wks with HFD showed a learning deficit in the acquisition phase as well as a negative memory effect in the probe test of MWM compared to ND [103]. Further, Jones et al. (2019) studied the effect of HFD-induced obesity and the apolipoprotein E4 (APOE4), both high risk factors for Alzheimer´s disease [3]. In contrast, they found no behavioral effect on spatial memory in the Barnes maze in APOE3 and APOE4 knock-in mice, after being on HFD for 12 wks. With regards to parameters in the MWM, Guo et al. (2020) used the distance during training and probe trial observing an obesity-induced memory reduction after 12 wks of HFD in mice [104]. In our study, we adducted this parameter rather to describe motor effort or activity. For evaluating memory impairments, the latency to find the target quadrant in the probe trial, directly after the acquisition phase, is used in most studies.

The effect of starting age and timing of HFD on the outcome of the study has been shown by Di Meco and Praticò (2019), where maternal fat-dense nutrition during gestation in mice had positive effects on brain health of the offspring in later life [97]. According to this, aged mice showed less tau pathology and caspase-3 activation in the brain. Additionally, they observed an improvement of learning and memory performance in the MWM test. Further, studies by Boitard et al. (2012, 2015) previously reported that mid-term exposure (8–12 wks) to HFD during adolescence, but not at adulthood, was linked to altered hippocampal function [105, 106], supporting the significance of starting age and duration of obesogenic diet for hippocampal-dependent memory. In line, other studies also proposed that HFD applied after the age of 8 wks did not induce harmful effects on spatial memory consolidation and spatial flexibility [23, 24].

Further differences in testing memory impairment are found relative to the applied test paradigm and the type of memory investigated. Gainey et al. (2016) described memory impairment using the Novel Object Recognition (NOR) and the Object Location Recognition (ORL) test already after one and three wks of HFD compared to low-fat diet in C57BL/6J mice [92]. The authors described this hippocampal-independent behavior which rapidly occurs after short-term HFD and normalizes with age. In line with our data, they state that hippocampal-sensitive memory develops not before long-term HFD. Next to the duration of the HFD, the composition can vary a lot between suppliers. However, most impact has the type of diet. A rather western diet, meaning a high-fat high-sugar diet (HFHSD), has been described to lead to cognitive impairment in humans and memory deficits in rodents [93]. In this study, even short-term HFHSD rapidly affected place recognition, but not object recognition memory in rats. Further, a dietary change back to ND recovered from this deficit. According to Jurdak et al. (2008) the brain seems to be more susceptive to sugar, leading rapidly to alterations in insulin and glucose metabolism causing cognitive impairment [102].

In conclusion, already mid-term HFD leads to a significant increase in body weight, which implicates further problems in general health. It has been shown that dietary change back to ND improves body weight and the appearance in mice. Here, long-term HFD alone does not cause learning deficits or spatial memory impairment in the Y maze and in the MWM test. However, long periods of excessive dietary fat intake increase microglial responses within the mediobasal hypothalamus, but not in the hippocampus showing neither neuroanatomical alterations nor dietary lipid accumulation, as is the case in the liver. HFD may have detrimental consequences for cognitive flexibility and mice may benefit from dietary change. Especially the type of diet—high-fat, high-sugar (sugar types) or western diet (containing high levels of fat and sugar)—and the precise diet composition are crucial for data interpretation and thus for therapeutic consequences of metabolic as well as cognitive diseases. In order to understand the correlation of different risk factors for cognitive impairment and Alzheimer’s disease, it is necessary to compare similar diets and test paradigms to investigate the underlying mechanisms.

## Supporting information

S1 Fig HFD does not lead to other general health issues. (A-E) SHIRPA analysis was used to estimate general health of mice after ND or HFD. (A) Long-term HFD did not lead to problems with eyes, (B) skin, (C) tail and (D) gait, or (E) fecal pellets. (F) Young and old mice as well as animals on HFD and ND showed a moderate motor reactivity indicated by the writhe reflex. Data are presented as mean values and error bars indicate SD; 8 wks ND n = 11, 4 wks HFD + 4 wks ND n = 12, 16 wks ND n = 24, 4 wks HFD + 12 wks ND n = 12, 12 wks HFD + 4 wks ND n = 7, 24 wks ND n = 30, 12 wks HFD + 12 wks ND n = 12, 24 wks HFD n = 10, 28 wks ND n = 12, 24 wks HFD + 4 wks ND n = 8.
(TIF)

S2 Fig Emotional state is not altered following long-term HFD. Depressive-like behavior following HFD was investigated applying the tail suspension test. (A) No effect of diet/dietary change was found on activity. (B) The latency to inactivity did not differ relative to diet. Data are presented as mean values and error bars indicate SD; 8 wks ND n = 12, 16 wks ND n = 24, 24 wks ND n = 30, 28 wks ND n = 12, 4 wks HFD + 4 wks ND n = 12, 12 wks HFD + 4 wks ND n = 7, 4 wks HFD + 12 wks ND n = 12, 12 wks HFD + 12 wks ND n = 12, 24 wks HFD n = 10, 24 wks HFD + 4 wks ND n = 9.
(TIF)

S3 Fig Upregulated hypothalamic *Iba1* expression following long-term HFD. Relative mRNA expression levels of *Iba1* in the hypothalamus, hippocampus and cortex of mice fed with ND or HFD for 24 wks. Data are presented as mean values and error bars indicate SD; 24 wks ND n = 3, 24 wks HFD n = 3; unpaired t test; * p < 0.05.
(TIF)

S4 Fig Long periods of HFD do not induce microglial activation in the frontal motor cortex. (A) Representative photomicrographs of double-labeled immunofluorescence staining for Iba1 (red) and NeuN (green) within the frontal motor cortex of male wild-type C57BL/6J mice fed with ND for eight wks, HFD for four wks followed by ND for four wks and ND or rather HFD for 24 wks. Nuclei were counterstained with DAPI. Scale bar corresponds to 25 μm. (B) Fluorescence intensity measurements of Iba1, (C) quantification of percentage of stained area with Iba1 and (E) NeuN and (D) number of Iba1-immunoreactive cells revealed no effect of HFD on microglial and neuronal morphology in the frontal cortex. Data are presented as mean values and error bars indicate SD; 8 wks ND n = 6, 4 wks HFD + 4 wks ND n = 6, 24 wks ND n = 12, 12 wks HFD + 12 wks ND n = 6, 24 wks HFD n = 6, 28 wks ND n = 6, 24 wks HFD + 4 wks ND n = 6.
(TIF)

S5 Fig HFD-induced obesity does not alter microglial density in the hippocampus. Microglial cell density did not change neither after diet/dietary change nor with age in CA1 (A) and CA3 (B) regions and dentate gyrus (C) of male wild-type C57BL/6J mice fed with ND for eight wks, HFD for four wks followed by ND for four wks and ND or rather HFD for 24 wks. Data are presented as mean values and error bars indicate SD; 8 wks ND n = 6, 4 wks HFD + 4 wks ND n = 6, 24 wks ND n = 12, 12 wks HFD + 12 wks ND n = 6, 24 wks HFD n = 6, 28 wks ND n = 6, 24 wks HFD + 4 wks ND n = 6.
(TIF)

S6 Fig Quantitative analysis of six morphological parameters of microglial cells reveals no microglial activation within the hippocampus and cortex after long-term HFD compared to age-matched control mice. Quantification of (A, G, M, S) microglial cell area, (B, H, N, T) cell perimeter, (C, I, O, U) cell convex hull area, (D, J, P, V) cell solidity, (E, K, Q, W) soma area and (F, L, R, X) skeleton length in CA1 and CA3 regions, dentate gyrus and frontal motor cortex of male wild-type C57BL/6J mice fed with ND for eight wks, HFD for four wks followed by ND for four wks, HFD for 12 wks followed by ND for 12 wks, ND or rather HFD for 24 wks, ND for 28 wks and HFD for 24 wks followed by ND for four wks. At least 180 cells per group were used for quantification. Data are presented as mean values and error bars indicate SD; 8 wks ND n = 6, 4 wks HFD + 4 wks ND n = 6, 24 wks ND n = 12, 12 wks HFD + 12 wks ND n = 6, 24 wks HFD n = 6, 28 wks ND n = 6, 24 wks HFD + 4 wks ND n = 6; One-way ANOVA; * p < 0.05.
(TIF)

S7 Fig Long-term HFD does not lead to lipid droplet accumulation within the hippocampus. Lipid droplets (A) per cell, (B) per tissue, (C) per cell area, (D) and per tissue area did not reveal any differences in lipid droplet accumulation neither after a long period of HFD nor with age in CA1, CA3 and dentate gyrus hippocampal regions. Data are presented as mean values and error bars indicate SD; 8 wks ND n = 4–5, 4 wks HFD + 4 wks ND n = 5–6, 24 wks ND n = 6, 24 wks HFD n = 6.
(TIF)

S1 Table Statistical analyses for all figures including statistical significances. The table shows relevant results of statistical tests.
(DOCX)

S2 Table Morphological parameters of microglial cells within the hippocampus and cortex in mice after HFD and/or ND exposure for varying weeks. (DOCX)

## Abbreviations

CNS: central nervous system
DAPI: 4′,6-diamidino-2-phenylindole
HFD: high-fat diet
HFHSD: high-fat high-sucrose diet
MWM: Morris Water Maze
ND: normal diet
NOR: Novel Object Recognition
ORL: Object Location Recognition
PBS: phosphate buffered saline
PFA: paraformaldehyde
POMC: proopiomelanocortin
SHIRPA: SmithKline Beecham, Harwell, Imperial College, Royal London Hospital, phenotype assessment
WKS: weeks
