# Supplementary figures and images for: Long-term diet-induced obesity does not lead to learning and memory impairment in adult mice
Source: PLoS One. 2021 Sep 29;16(9):e0257921. doi: 10.1371/journal.pone.0257921 (PMC8480843; doi:10.1371/journal.pone.0257921)

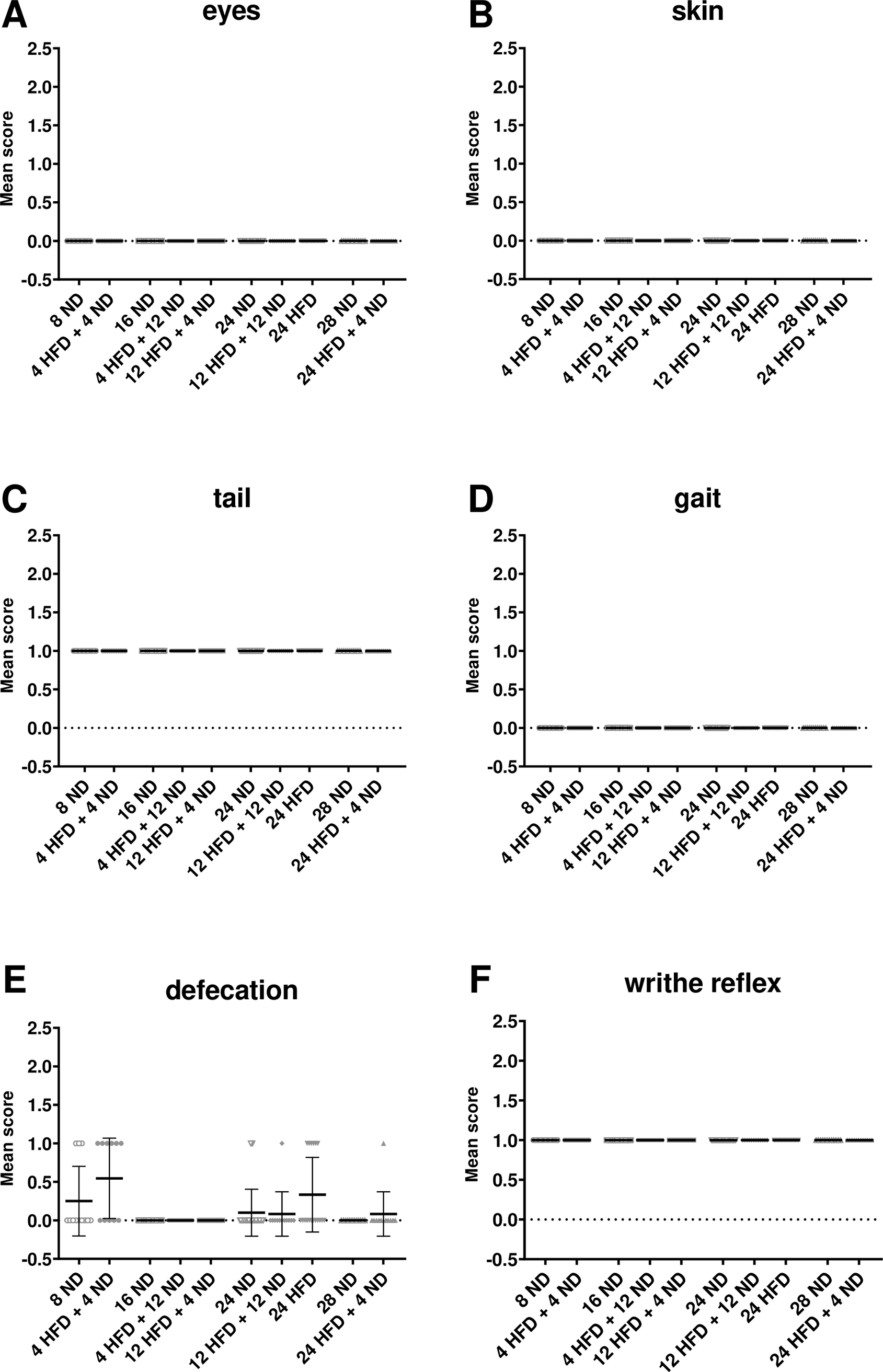

Supplement: S1 Fig — (A-E) SHIRPA analysis was used to estimate general health of mice after ND or HFD. (A) Long-term HFD did not lead to problems with eyes, (B) skin, (C) tail and (D) gait, or (E) fecal pellets. (F) Young and old mice as well as animals on HFD and ND showed a moderate motor reactivity indicated by the writhe reflex. Data are presented as mean values and error bars indicate SD; 8 wks ND n = 11, 4 wks HFD + 4 wks ND n = 12, 16 wks ND n = 24, 4 wks HFD + 12 wks ND n = 12, 12 wks HFD + 4 wks ND n = 7, 24 wks ND n = 30, 12 wks HFD + 12 wks ND n = 12, 24 wks HFD n = 10, 28 wks ND n = 12, 24 wks HFD + 4 wks ND n = 8. (TIF) [file pone.0257921.s001.tif]

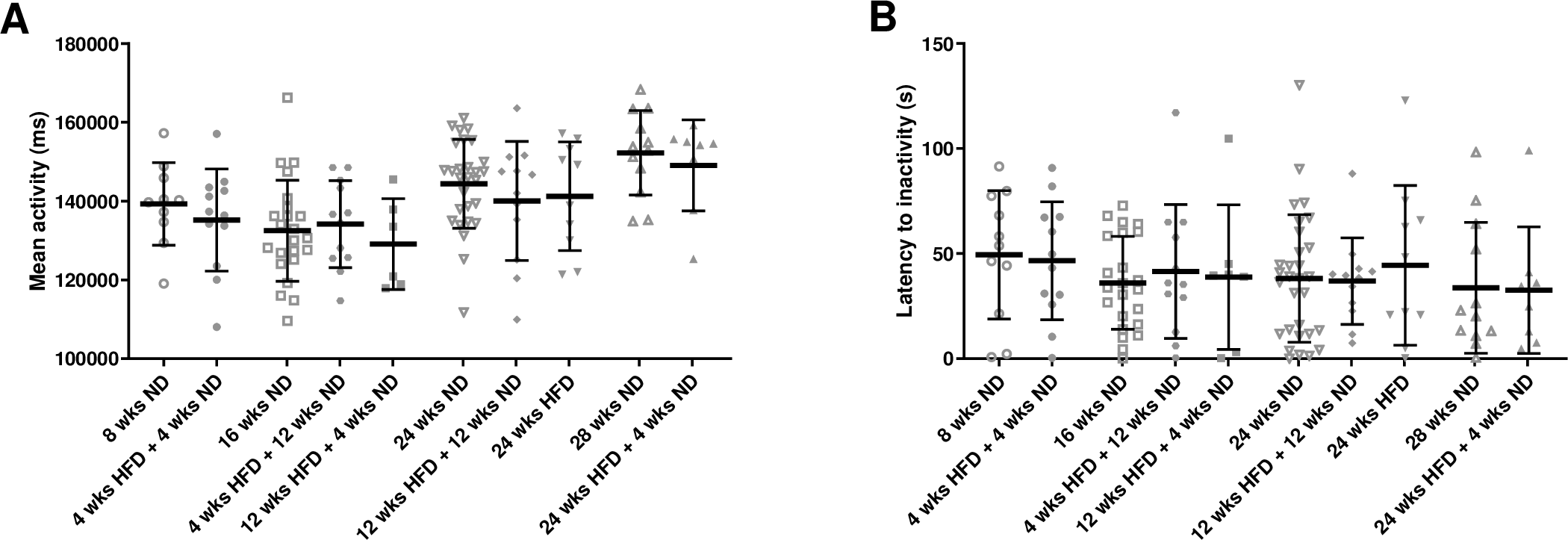

Supplement: S2 Fig — Depressive-like behavior following HFD was investigated applying the tail suspension test. (A) No effect of diet/dietary change was found on activity. (B) The latency to inactivity did not differ relative to diet. Data are presented as mean values and error bars indicate SD; 8 wks ND n = 12, 16 wks ND n = 24, 24 wks ND n = 30, 28 wks ND n = 12, 4 wks HFD + 4 wks ND n = 12, 12 wks HFD + 4 wks ND n = 7, 4 wks HFD + 12 wks ND n = 12, 12 wks HFD + 12 wks ND n = 12, 24 wks HFD n = 10, 24 wks HFD + 4 wks ND n = 9. (TIF) [file pone.0257921.s002.tif]

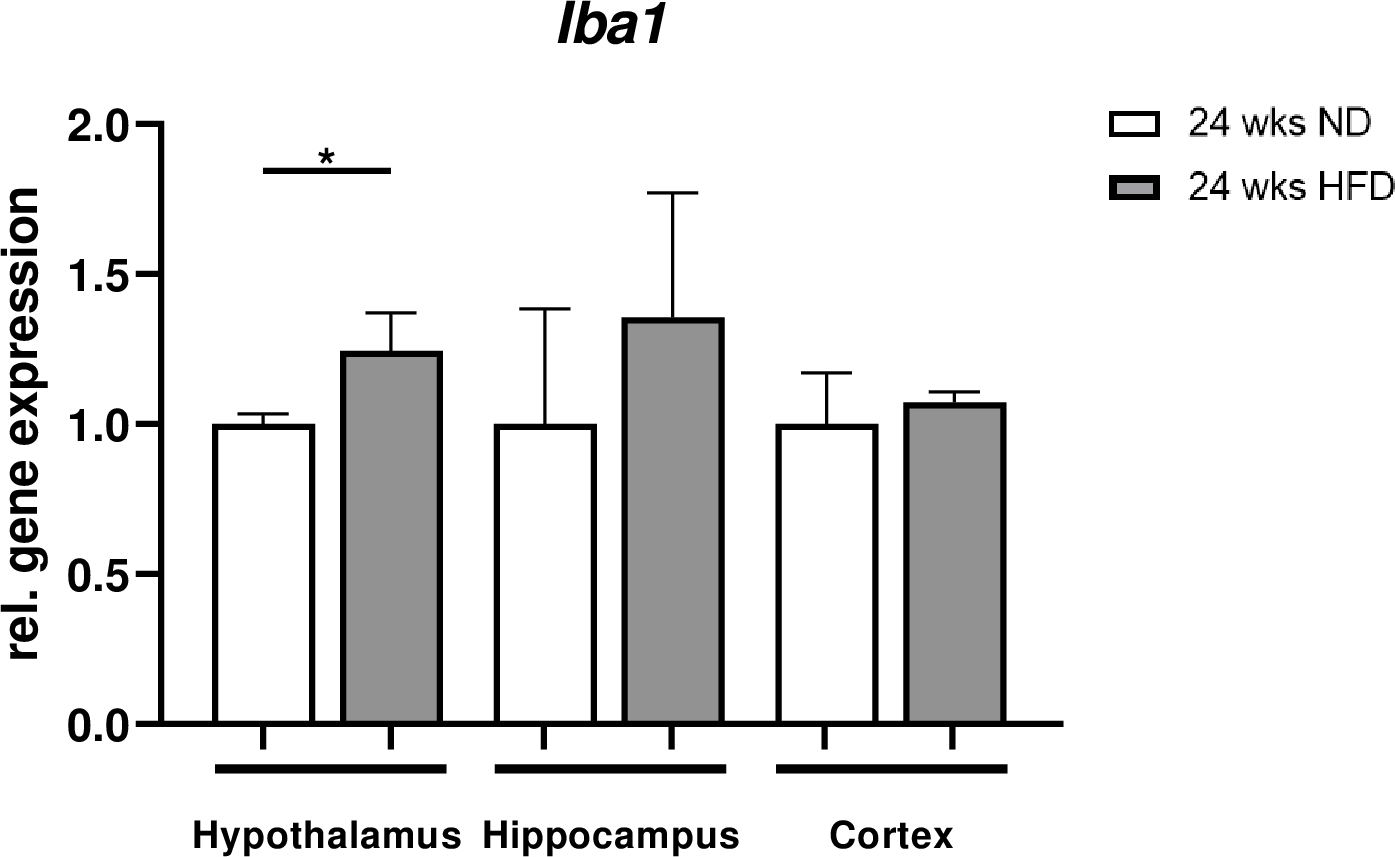

Supplement: S3 Fig — Relative mRNA expression levels of Iba1 in the hypothalamus, hippocampus and cortex of mice fed with ND or HFD for 24 wks. Data are presented as mean values and error bars indicate SD; 24 wks ND n = 3, 24 wks HFD n = 3; unpaired t test; * p < 0.05. (TIF) [file pone.0257921.s003.tif]

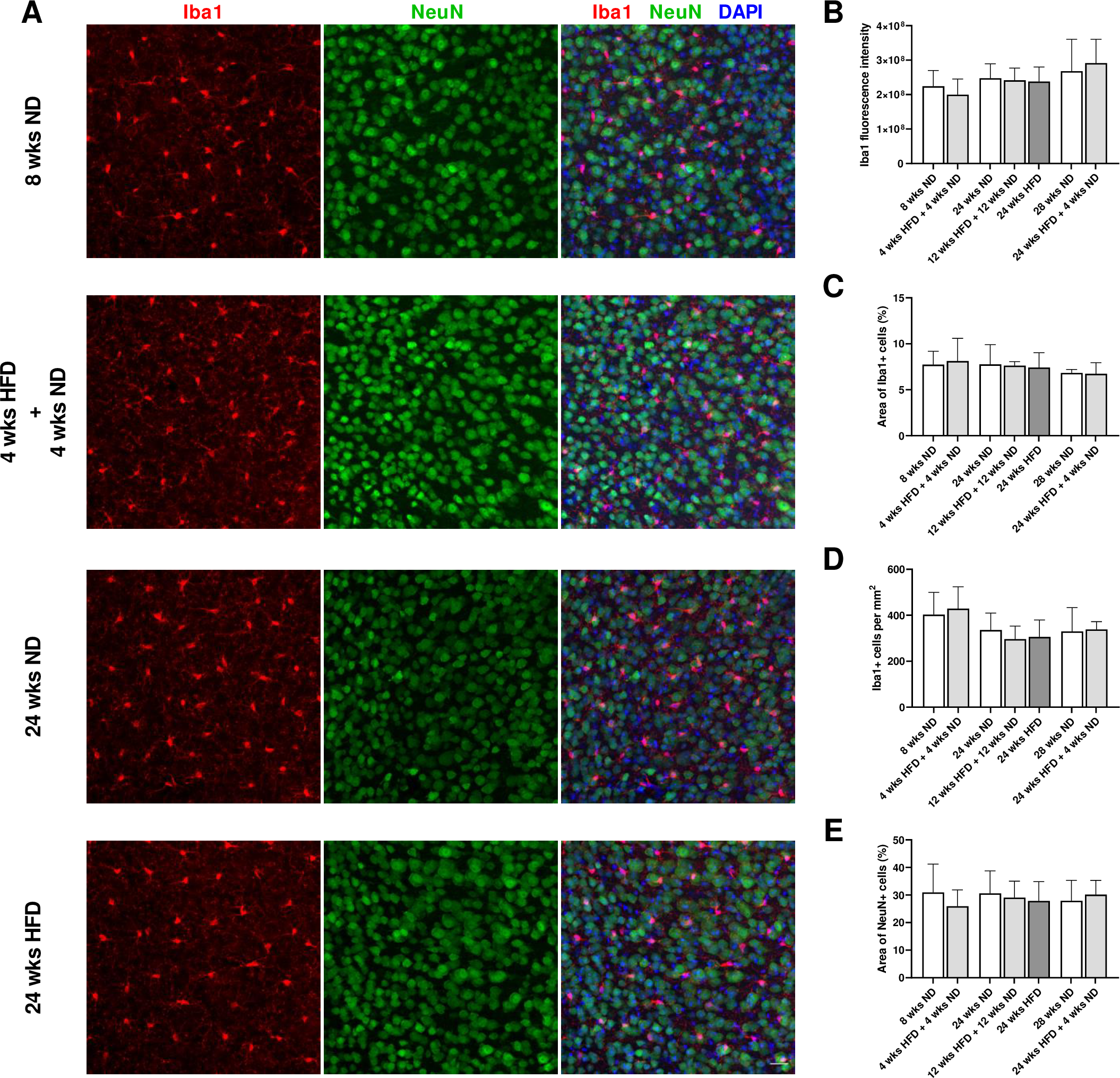

Supplement: S4 Fig — (A) Representative photomicrographs of double-labeled immunofluorescence staining for Iba1 (red) and NeuN (green) within the frontal motor cortex of male wild-type C57BL/6J mice fed with ND for eight wks, HFD for four wks followed by ND for four wks and ND or rather HFD for 24 wks. Nuclei were counterstained with DAPI. Scale bar corresponds to 25 μm. (B) Fluorescence intensity measurements of Iba1, (C) quantification of percentage of stained area with Iba1 and (E) NeuN and (D) number of Iba1-immunoreactive cells revealed no effect of HFD on microglial and neuronal morphology in the frontal cortex. Data are presented as mean values and error bars indicate SD; 8 wks ND n = 6, 4 wks HFD + 4 wks ND n = 6, 24 wks ND n = 12, 12 wks HFD + 12 wks ND n = 6, 24 wks HFD n = 6, 28 wks ND n = 6, 24 wks HFD + 4 wks ND n = 6. (TIF) [file pone.0257921.s004.tif]

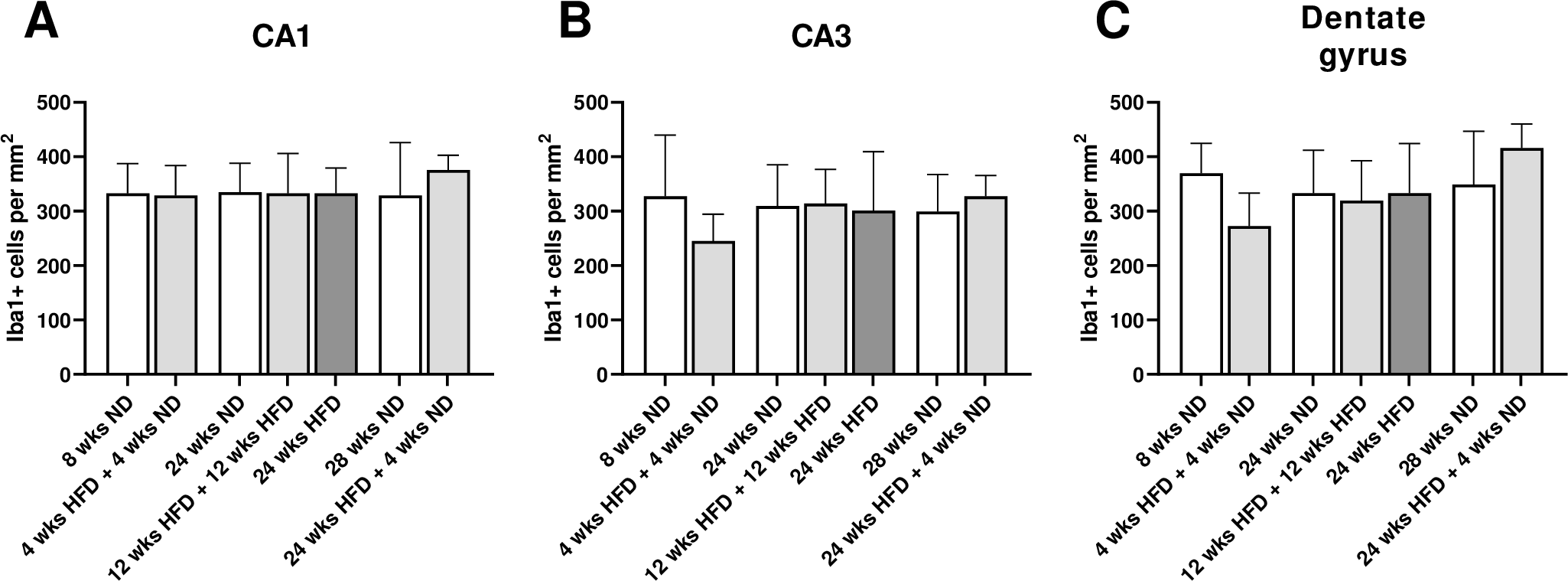

Supplement: S5 Fig — Microglial cell density did not change neither after diet/dietary change nor with age in CA1 (A) and CA3 (B) regions and dentate gyrus (C) of male wild-type C57BL/6J mice fed with ND for eight wks, HFD for four wks followed by ND for four wks and ND or rather HFD for 24 wks. Data are presented as mean values and error bars indicate SD; 8 wks ND n = 6, 4 wks HFD + 4 wks ND n = 6, 24 wks ND n = 12, 12 wks HFD + 12 wks ND n = 6, 24 wks HFD n = 6, 28 wks ND n = 6, 24 wks HFD + 4 wks ND n = 6. (TIF) [file pone.0257921.s005.tif]

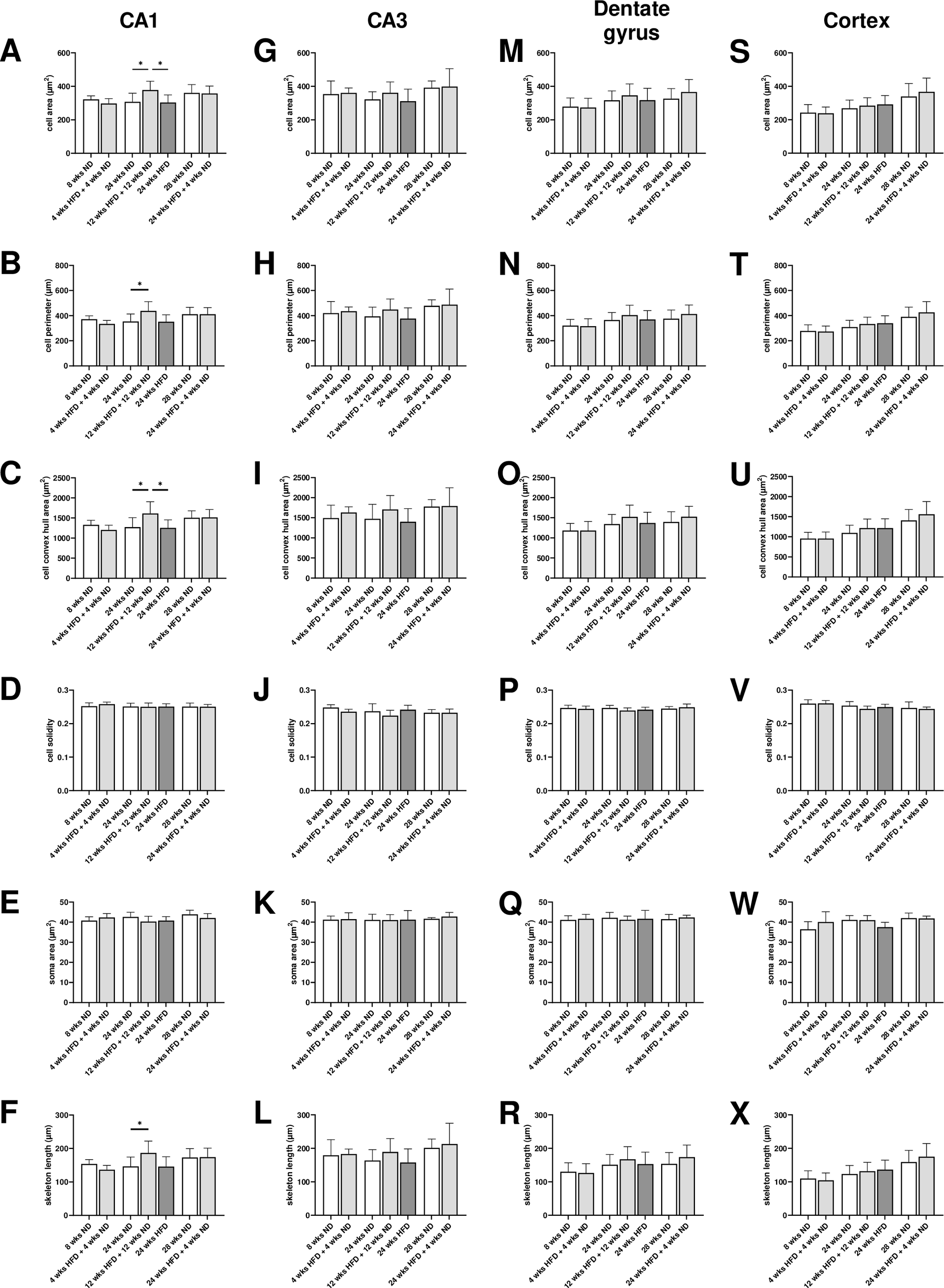

Supplement: S6 Fig — Quantification of (A, G, M, S) microglial cell area, (B, H, N, T) cell perimeter, (C, I, O, U) cell convex hull area, (D, J, P, V) cell solidity, (E, K, Q, W) soma area and (F, L, R, X) skeleton length in CA1 and CA3 regions, dentate gyrus and frontal motor cortex of male wild-type C57BL/6J mice fed with ND for eight wks, HFD for four wks followed by ND for four wks, HFD for 12 wks followed by ND for 12 wks, ND or rather HFD for 24 wks, ND for 28 wks and HFD for 24 wks followed by ND for four wks. At least 180 cells per group were used for quantification. Data are presented as mean values and error bars indicate SD; 8 wks ND n = 6, 4 wks HFD + 4 wks ND n = 6, 24 wks ND n = 12, 12 wks HFD + 12 wks ND n = 6, 24 wks HFD n = 6, 28 wks ND n = 6, 24 wks HFD + 4 wks ND n = 6; One-way ANOVA; * p < 0.05. (TIF) [file pone.0257921.s006.tif]

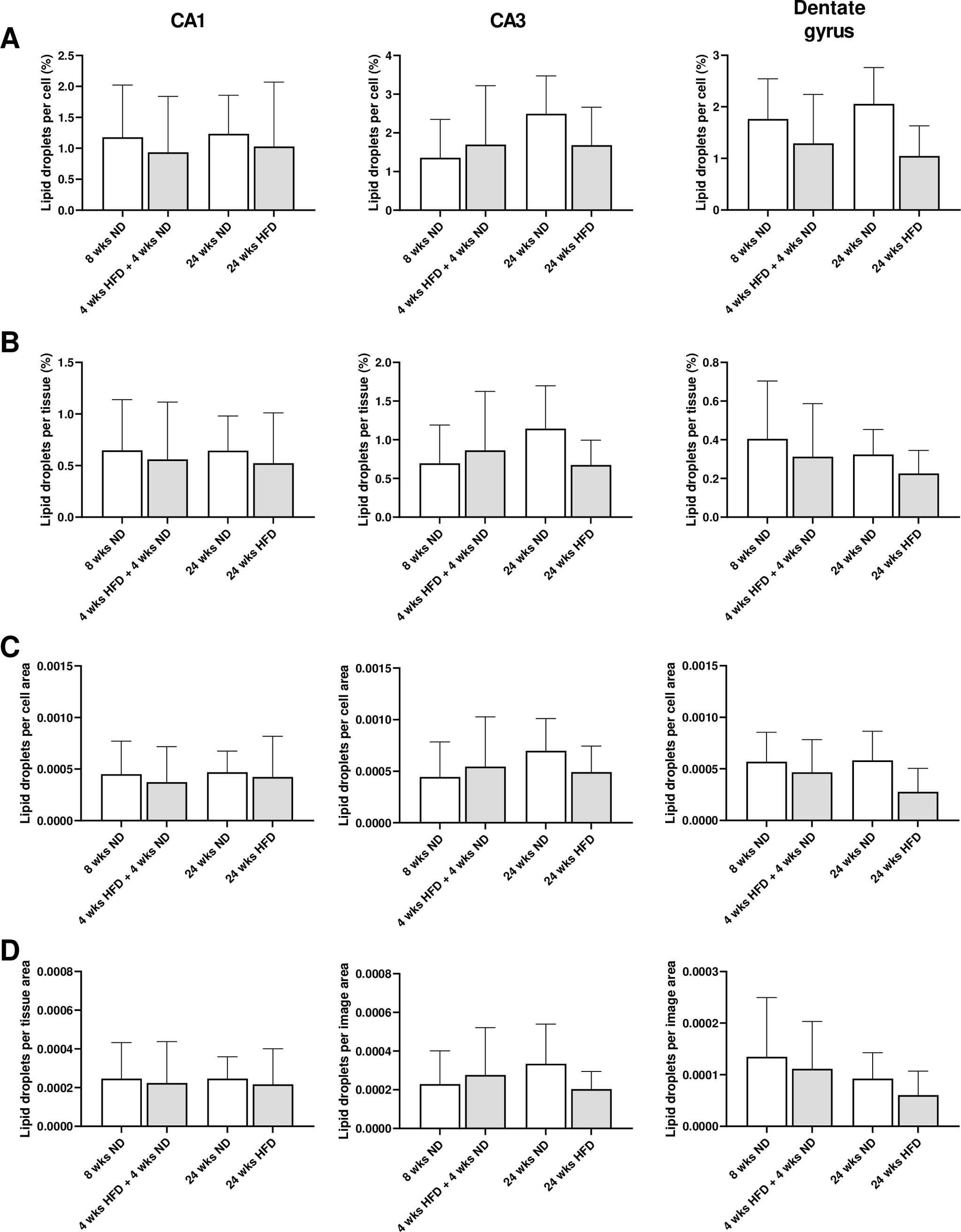

Supplement: S7 Fig — Lipid droplets (A) per cell, (B) per tissue, (C) per cell area, (D) and per tissue area did not reveal any differences in lipid droplet accumulation neither after a long period of HFD nor with age in CA1, CA3 and dentate gyrus hippocampal regions. Data are presented as mean values and error bars indicate SD; 8 wks ND n = 4–5, 4 wks HFD + 4 wks ND n = 5–6, 24 wks ND n = 6, 24 wks HFD n = 6. (TIF) [file pone.0257921.s007.tif]
