# Supplementary material for: Long-term diet-induced obesity does not lead to learning and memory impairment in adult mice
Source: PLoS One. 2021 Sep 29;16(9):e0257921. doi: 10.1371/journal.pone.0257921 (PMC8480843; doi:10.1371/journal.pone.0257921)
Supplement: S1 Table — The table shows relevant results of statistical tests. (DOCX) [file pone.0257921.s008.docx]

**S1 Table**. Statistical analyses for all figures including statistical significances. The table shows relevant results of statistical tests.

1

2

3

4

5

6

7

8

9

10

11

12

13

14

15

16

17

18

19

20

21

22

23

24

25

26

27

28

29

30

31

32

33

34

35

36

37

38

39

40

41

42

43

| Figure | Statistical significances | Statistical test | p-value |
| --- | --- | --- | --- |
| 1C | 16 wks ND vs. 12 wks HFD + 4 wks ND ****, 4 wks HFD + 12 wks ND vs. 12 wks HFD + 4 wks ND ****  24 wks ND vs. 24 wks HFD ****, 12 wks HFD + 12 wks ND vs. 24 wks HFD ****  28 wks ND vs. 24 wks HFD + 4 wks ND ****  24 wks HFD vs. 24 wks HFD + 4 wks ND **  8 wks ND vs. 16, 28 wks ND ***, 8 wks ND vs. 24 wks ND ****  4 wks HFD + 4 wks ND vs. 12 wks HFD + 4 wks ND, 24 wks HFD + 4 wks ND ****, 12 wks HFD + 4 wks ND vs. 24 wks HFD + 4 wks ND *  4 wks HFD + 12 wks ND vs. 12 wks HFD + 12 wks ND **** | One-way ANOVA  Welch-ANOVA  Unpaired t test  Unpaired t test  One-way ANOVA  One-way ANOVA  Unpaired t test | < 0.0001  < 0.0001  < 0.0001  0.0024  < 0.0001  < 0.0001  < 0.0001 |
| 1D | 16 wks ND vs. 12 wks HFD + 4 wks ND ***, 4 wks HFD + 12 wks ND vs. 12 wks HFD + 4 wks ND ****  24 wks ND vs. 24 wks HFD ****, 12 wks HFD + 12 wks ND vs. 24 wks HFD ****  28 wks ND vs. 24 wks HFD + 4 wks ND ****  24 wks HFD vs. 24 wks HFD + 4 wks ND **  8 wks ND vs. 16, 24, 28 wks ND ****, 16 wks ND vs. 24, 28 wks ND ***  4 wks HFD + 4 wks ND vs. 12 wks HFD + 4 wks ND *, 4 wks HFD + 4 wks ND vs. 24 wks HFD + 4 wks ND ****  4 wks HFD + 12 wks ND vs. 12 wks HFD + 12 wks ND *** | Kruskal-Wallis test  Welch-ANOVA  Unpaired t test  Unpaired t test  One-way ANOVA  Kruskal-Wallis test  Welch’s t test | < 0.0001  < 0.0001  < 0.0001  < 0.0001  < 0.0001  < 0.0001  0.0003 |
| 1F | 24 wks ND vs. 24 wks HFD **  24 wks HFD vs. 24 wks HFD + 4 wks ND ****  28 wks ND vs. 24 wks HFD + 4 wks ND ** | Mann-Whitney test  Unpaired t test  Mann-Whitney test | 0.0022  < 0.0001  0.0022 |
| 2A | 24 wks ND vs. 24 wks HFD *, 12 wks HFD + 12 wks ND vs. 24 wks HFD * | Kruskal-Wallis test | 0.0124 |
| 2B | 24 wks ND vs. 24 wks HFD ****, 12 wks HFD + 12 wks ND vs. 24 wks HFD ****  28 wks ND vs. 24 wks HFD + 4 wks ND *  4 wks HFD + 4 wks ND vs. 24 wks HFD + 4 wks ND **, 12 wks HFD + 4 wks ND vs. 24 wks HFD + 4 wks ND ** | Kruskal-Wallis test  Mann-Whitney test  Kruskal-Wallis test | < 0.0001  0.0036  0.0012 |
| 3C | 24 wks ND vs. 12 wks HFD + 12 wks ND *, 12 wks HFD + 12 wks ND vs. 24 wks HFD **  8 wks ND vs. 24 wks ND **, 8 wks ND vs. 28 wks ND *, 16 wks ND vs. 24 wks ND ***, 16 wks ND vs. 28 wks ND **  4 wks HFD + 4 wks ND vs. 24 wks HFD + 4 wks ND * | One-way ANOVA  Kruskal-Wallis test  One-way ANOVA | 0.0021  < 0.0001  0.0129 |
| 3D | 16 wks ND vs. 12 wks HFD + 4 wks ND ****, 4 wks HFD + 12 wks ND vs. 12 wks HFD + 4 wks ND ****  24 wks ND vs. 24 wks HFD ****, 12 wks HFD + 12 wks ND vs. 24 wks HFD ****  28 wks ND vs. 24 wks HFD + 4 wks ND ****  8 wks ND vs. 16, 28 wks ND ***, 8 wks ND vs. 24 wks ND ****  4 wks HFD + 4 wks ND vs. 12 wks HFD + 4 wks ND, 24 wks HFD + 4 wks ND ****, 12 wks HFD + 4 wks ND vs. 24 wks HFD + 4 wks ND **  4 wks HFD + 12 wks ND vs. 12 wks HFD + 12 wks ND *** | One-way ANOVA  Welch-ANOVA  Unpaired t test  One-way ANOVA  One-way ANOVA  Unpaired t test | < 0.0001  < 0.0001  < 0.0001  < 0.0001  < 0.0001  0.0002 |
| 4A | 1🡪3: 16 wks ND ****, 4 wks HFD + 12 wks ND ****, 12 wks HFD + 4 wks ND *  Day 1: 12 wks HFD + 12 wks ND vs. 24 wks HFD **; Day 2: 12 wks HFD + 12 wks ND vs. 24 wks HFD *; Day 3: 24 wks ND vs. 24 wks HFD *  1🡪3: 24 wks ND ****, 12 wks HFD + 12 wks ND ***, 24 wks HFD ***  1🡪3: 28 wks ND *, 24 wks HFD + 4 wks ND ***  Day 3: 8 wks ND vs. 24, 28 wks ND *  1🡪3: 16 wks ND ****, 24 wks ND ****, 28 wks ND *  Day 1: 4 wks HFD + 4 wks ND vs. 24 wks HFD + 4 wks ND ****, 12 wks HFD + 4 wks ND vs. 24 wks HFD + 4 wks ND *  1🡪3: 12 wks HFD + 4 wks ND *, 24 wks HFD + 4 wks ND *** | Two-way ANOVA  Two-way ANOVA  Two-way ANOVA  Two-way ANOVA  Two-way ANOVA | Day: < 0.0001  Diet: ns  Interaction: ns  Day: < 0.0001  Diet: 0.0003  Interaction: 0.0432  Day: < 0.0001  Diet: ns  Interaction: ns  Day: < 0.0001  Age: ns  Interaction: ns  Day: < 0.0001  Age: ns  Interaction: 0.0001 |
| 4B  44  45  46  47  48  49  50  51  52  53  54  55  56  57  58  59  60  61  62  63  64  65  66  67  68  69  70  71  72  73  74  75  76  77  78  79  80  81  82  83  84  85  86  87  88  89 | 1🡪3: 8 wks ND **, 4 wks HFD + 4 wks ND **  1🡪3: 16 wks ND ****, 4 wks HFD + 12 wks ND ***, 12 wks HFD + 4 wks ND *  Day 1: 12 wks HFD + 12 wks ND vs. 24 wks ND *  1🡪3: 24 wks ND ****, 24 wks HFD **  1🡪3: 28 wks ND ****, 24 wks HFD + 4 wks ND ****  Day 3: 8 wks ND vs. 16 wks ND *  1🡪3: 8 wks ND **, 16, 24 wks ND ****, 28 wks ND ***  Day 1: 4 wks HFD + 4 wks ND vs. 12 wks HFD + 4 wks ND *, 12 wks HFD + 4 wks ND vs. 24 wks HFD + 4 wks ND **  1🡪3: 4 wks HFD + 4 wks ND **, 12 wks HFD + 4 wks ND *, 24 wks HFD + 4 wks ND ** | Two-way ANOVA  Two-way ANOVA  Two-way ANOVA  Two-way ANOVA  Two-way ANOVA  Two-way ANOVA | Day: < 0.0001  Diet: ns  Interaction: ns  Day: < 0.0001  Diet: ns  Interaction: ns  Day: < 0.0001  Diet: ns  Interaction: ns  Day: < 0.0001  Diet: ns  Interaction: ns  Day: < 0.0001  Age: ns  Interaction: ns  Day: < 0.0001  Age: 0.0021  Interaction: 0.0036 |
| 4C | 1🡪3: 8 wks ND *  1🡪3: 16 wks ND ****, 4 wks HFD + 12 wks ND ***, 12 wks HFD + 4 wks ND **  1🡪3: 24 wks ND ****, 12 wks HFD + 12 wks ND *, 24 wks HFD **  1🡪3: 28 wks ND ****, 24 wks HFD + 4 wks ND ***  Day 1: 16 wks ND vs. 24 wks ND *; Day 3: 8 wks ND vs. 24, 28 wks ND *  1🡪3: 8 wks ND *, 16, 24, 28 wks ND ****  Day 1: 12 wks HFD + 4 wks ND vs. 24 wks HFD + 4 wks ND *  1🡪3: 12 wks HFD + 4 wks ND **, 24 wks HFD + 4 wks ND *** | Two-way ANOVA  Two-way ANOVA  Two-way ANOVA  Two-way ANOVA  Two-way ANOVA  Two-way ANOVA | Day: 0.0009  Diet: ns  Interaction: ns  Day: < 0.0001  Diet: ns  Interaction: ns  Day: < 0.0001  Diet: ns  Interaction: 0.0145  Day: < 0.0001  Diet: ns  Interaction: ns  Day: < 0.0001  Age: < 0.0001  Interaction: < 0.0001  Day: 0.0067  Age: 0.0144  Interaction: ns |
| 4D | Day 1: 8 wks ND vs. 4 wks HFD + 4 wks ND *; Day 4: 8 wks ND vs. 4 wks HFD + 4 wks ND *  1🡪4: 8 wks ND *  Day 1: 16 wks ND vs. 4 wks HFD + 12 wks ND *  Day 1: 24 wks ND vs. 24 wks HFD **, 12 wks HFD + 12 wks ND vs. 24 wks HFD ***  Day 1: 8 wks ND vs. 16 wks ND ****, 16 wks ND vs. 24 wks ND **; Day 2: 8 wks ND vs. 16 wks ND *  1🡪4: 8 wks ND * | Two-way ANOVA  Two-way ANOVA  Two-way ANOVA  Two-way ANOVA | Day: ns  Diet: ns  Interaction: 0.0015  Day: 0.0136  Diet: ns  Interaction: ns  Day: ns  Diet: ns  Interaction: 0.0059  Day: ns  Age: 0.0155  Interaction: 0.0010 |
| 4E  90  91  92  93  94  95  96  97  98  99  100  101  102  103  104  105  106  107  108  109  110  111  112  113  114  115  116  117  118  119  120  121  122  123  124  125  126  127  128  129  130  131  132  133  134  135 | Day 4: 8 wks ND vs. 4 wks HFD + 4 wks ND *  1🡪4: 4 wks HFD + 4 wks ND **  1🡪4: 16 wks ND **, 12 wks HFD + 4 wks ND **  1🡪4: 24 wks ND ****, 12 wks HFD + 12 wks ND *, 24 wks HFD **  1🡪4: 28 wks ND **, 24 wks HFD + 4 wks ND **  Day 1: 8 wks ND vs. 16, 24, 28 wks ND **; Day 2: 8 wks ND vs. 16 wks ND *; Day 4: 8 wks ND vs. 16 wks ND **  1🡪4: 16, 28 wks ND **, 24 wks ND ****  Day 1: 4 wks HFD + 4 wks ND vs. 12 wks HFD + 4 wks ND *  1🡪4: 4 wks HFD + 4 wks ND **, 12 wks HFD + 4 wks ND **, 24 wks HFD + 4 wks ND ** | Two-way ANOVA  Two-way ANOVA  Two-way ANOVA  Two-way ANOVA  Two-way ANOVA  Two-way ANOVA | Day: < 0.0001  Diet: ns  Interaction: 0.0002  Day: < 0.0001  Diet: ns  Interaction: ns  Day: < 0.0001  Diet: ns  Interaction: ns  Day: < 0.0001  Diet: ns  Interaction: ns  Day: < 0.0001  Age: 0.0006  Interaction: 0.0101  Day: < 0.0001  Age: 0.0003  Interaction: ns |
| 4F | 1🡪4: 4 wks HFD + 4 wks ND **  1🡪4: 16 wks ND ***, 4 wks HFD + 12 wks ND **, 12 wks HFD + 4 wks ND ****  1🡪4: 24 wks ND ****, 12 wks HFD + 12 wks ND **, 24 wks HFD ***  1🡪4: 28 wks ND **, 24 wks HFD + 4 wks ND **  Day 4: 8 wks ND vs. 16 wks ND *  1🡪4: 16 wks ND ***, 24 wks ND ****, 28 wks ND **  Day 1: 4 wks HFD + 4 wks ND vs. 12 wks HFD + 4 wks ND *  Day 3: 4 wks HFD + 4 wks ND vs. 12 wks HFD + 4 wks ND **, 12 wks HFD + 4 wks ND vs. 24 wks HFD + 4 wks ND **  1🡪4: 4 wks HFD + 4 wks ND **, 12 wks HFD + 4 wks ND ***, 24 wks HFD + 4 wks ND ** | Two-way ANOVA  Two-way ANOVA  Two-way ANOVA  Two-way ANOVA  Two-way ANOVA  Two-way ANOVA | Day: < 0.0001  Diet: ns  Interaction: 0.0346  Day: < 0.0001  Diet: ns  Interaction: 0.0336  Day: < 0.0001  Diet: ns  Interaction: ns  Day: < 0.0001  Diet: ns  Interaction: ns  Day: < 0.0001  Age: ns  Interaction: 0.0046  Day: < 0.0001  Age: 0.0488  Interaction: 0.0141 |
| 5A | Target quadrant – opposite quadrant: 8 wks ND *, 4 wks HFD + 4 wks ND ***  Target quadrant – opposite quadrant: 16 wks ND ****, 4 wks HFD + 12 wks ND ****, 12 wks HFD + 4 wks ND ***  Target quadrant – opposite quadrant: 24 wks ND ****, 12 wks HFD + 12 wks ND ***, 24 wks HFD ****  136  137  138  139  140  141  142  143  144  145  146  147  148  149  150  151  152  153  154  155  156  157  158  159  160  161  162  163  164  165  166  167  168  169  170  171  172  173  174  175  176  177  178  179  180  181  Target quadrant – opposite quadrant: 28 wks ND ****, 24 wks HFD + 4 wks ND ****  Target quadrant: 8 wks ND vs. 28 wks ND *  Target quadrant: 4 wks HFD + 4 wks ND vs. 24 wks HFD + 4 wks ND ***  Opposite quadrant: 4 wks HFD + 4 wks ND vs. 24 wks HFD + 4 wks ND * | Two-way ANOVA  Two-way ANOVA  Two-way ANOVA  Two-way ANOVA  Two-way ANOVA  Two-way ANOVA | Quadrant: < 0.0001  Diet: ns  Interaction: ns  Diet: ns  Quadrant: < 0.0001  Interaction: ns  Quadrant: < 0.0001  Diet: ns  Interaction: ns  Diet: ns  Quadrant: < 0.0001  Interaction: ns  Quadrant: < 0.0001  Age: ns  Interaction: 0.0191  Quadrant: < 0.0001  Age: ns  Interaction: 0.0021 |
| 5C | 24 wks ND vs. 24 wks HFD **, 12 wks HFD + 12 wks ND vs. 24 wks HFD ***  8 wks ND vs. 16 wks ND **, 8 wks ND vs. 28 wks ND ****  4 wks HFD + 4 wks ND vs. 12 wks HFD + 4 wks ND **, 4 wks HFD + 4 wks ND vs. 24 wks HFD + 4 wks ND *** | One-way ANOVA  Kruskal-Wallis test  One-way ANOVA | 0.0006  0.0002  0.0002 |
| 5D | 16 wks ND vs. 12 wks HFD + 4 wks ND ****, 4 wks HFD + 12 wks ND vs. 12 wks HFD + 4 wks ND ****  24 wks ND vs. 24 wks HFD ****, 12 wks HFD + 12 wks ND vs. 24 wks HFD ****  28 wks ND vs. 24 wks HFD + 4 wks ND ****  24 wks HFD vs. 24 wks HFD + 4 wks ND *  8 wks ND vs. 16 wks ND ***, 8 wks ND vs. 24 wks ND ****, 8 wks ND vs. 28 wks ND **  4 wks HFD + 4 wks ND vs. 12 wks HFD + 4 wks ND, 24 wks HFD + 4 wks ND ****, 12 wks HFD + 4 wks ND vs. 24 wks HFD + 4 wks ND *  4 wks HFD + 12 wks ND vs. 12 wks HFD + 12 wks ND *** | One-way ANOVA  Welch-ANOVA  Unpaired t test  Unpaired t test  One-way ANOVA  One-way ANOVA  Unpaired t test | < 0.0001  < 0.0001  < 0.0001  0.0268  < 0.0001  < 0.0001  0.0001 |
| 6A | Target quadrant – opposite quadrant: 4 wks HFD + 4 wks ND **  Target quadrant – opposite quadrant: 16 wks ND **, 4 wks HFD + 12 wks ND ***, 12 wks HFD + 4 wks ND **  Target quadrant – opposite quadrant: 24 wks ND ****, 24 wks HFD ****  Target quadrant: 24 wks ND vs. 24 wks HFD ***, 12 wks HFD + 12 wks ND vs. 24 wks HFD ****  Opposite quadrant: 24 wks ND vs. 24 wks HFD **, 12 wks HFD + 12 wks ND vs. 24 wks HFD **  Target quadrant – opposite quadrant: 28 wks ND **, 24 wks HFD + 4 wks ND **  Target quadrant: 4 wks HFD + 4 wks ND vs. 12 wks HFD + 4 wks ND **, 4 wks HFD + 4 wks ND vs. 24 wks HFD + 4 wks ND ** | Two-way ANOVA  Two-way ANOVA  Two-way ANOVA  Two-way ANOVA  Two-way ANOVA | Quadrant: 0.0007  Diet: ns  Interaction: ns  Quadrant: < 0.0001  Diet: ns  Interaction: ns  Quadrant: < 0.0001  Diet: ns  Interaction: < 0.0001  Quadrant: < 0.0001  Diet: ns  Interaction: ns  Quadrant: < 0.0001  Age: ns  Interaction: 0.0153 |
| 6C | 24 wks ND vs. 24 wks HFD **, 12 wks HFD + 12 wks ND vs. 24 wks HFD *  8 wks ND vs. 16, 28 wks ND ****, 8 wks ND vs. 24 wks ND *, 16 wks ND vs. 24 wks ND **, 24 wks ND vs. 28 wks ND **  4 wks HFD + 4 wks ND vs. 12 wks HFD + 4 wks ND ****, 4 wks HFD + 4 wks ND vs. 24 wks HFD + 4 wks ND **** | One-way ANOVA  One-way ANOVA  One-way ANOVA | 0.0016  < 0.0001  < 0.0001 |
| 6D | 16 wks ND vs. 12 wks HFD + 4 wks ND *, 4 wks HFD + 12 wks ND vs. 12 wks HFD + 4 wks ND * | Kruskal-Wallis test | 0.0164 |
| 6E | 16 wks ND vs. 12 wks HFD + 4 wks ND *, 4 wks HFD + 12 wks ND vs. 12 wks HFD + 4 wks ND ** | Kruskal-Wallis test | 0.0052 |
| 7B | 24 wks ND vs. 24 wks HFD **  24 wks HFD vs. 24 wks HFD + 4 wks ND ** | Welch-ANOVA  Unpaired t test | 0.0069  0.0047 |
| 7C | 24 wks ND vs. 24 wks HFD ***  24 wks HFD vs. 24 wks HFD + 4 wks ND * | One-way ANOVA  Unpaired t test | 0.0003  0.0346 |
| 7D | 4 wks HFD + 4 wks ND vs. 24 wks HFD + 4 wks ND ** | Unpaired t test | 0.0016 |
| S2A | 16 wks ND vs. 24 wks ND **, 16 wks ND vs. 28 wks ND ****  12 wks HFD + 4 wks ND vs. 24 wks HFD + 4 wks ND * | One-way ANOVA  Kruskal-Wallis test | < 0.0001  0.0188 |
| S3 | 24 wks ND vs. 24 wks HFD * (Hypothalamus) | Unpaired t test | 0.0328 |
| S6A | 24 wks ND vs. 12 wks HFD + 12 wks ND *, 12 wks HFD + 12 wks ND vs. 24 wks HFD * | One-way ANOVA | 0.0240 |
| S6B | 24 wks ND vs. 12 wks HFD + 12 wks ND * | One-way ANOVA | 0.0305 |
| S6C | 24 wks ND vs. 12 wks HFD + 12 wks ND *, 12 wks HFD + 12 wks ND vs. 24 wks HFD * | One-way ANOVA | 0.0226 |
| S6F | 24 wks ND vs. 12 wks HFD + 12 wks ND * | One-way ANOVA | 0.0349 |
| S6S | 8 wks ND vs. 28 wks ND **, 24 wks ND vs. 28 wks ND *  4 wks HFD + 4 wks ND vs. 24 wks HFD + 4 wks ND ** | One-way ANOVA  Unpaired t test | 0.0203  0.0056 |
| S6T | 8 wks ND vs. 28 wks ND *  4 wks HFD + 4 wks ND vs. 24 wks HFD + 4 wks ND ** | One-way ANOVA  Unpaired t test | 0.0088  0.0031 |
| S6U | 8 wks ND vs. 28 wks ND **, 24 wks ND vs. 28 wks ND *  4 wks HFD + 4 wks ND vs. 24 wks HFD + 4 wks ND ** | One-way ANOVA  Unpaired t test | 0.0029  0.0021 |
| S6X | 8 wks ND vs. 28 wks ND *, 24 wks ND vs. 28 wks ND *  4 wks HFD + 4 wks ND vs. 24 wks HFD + 4 wks ND ** | One-way ANOVA  Unpaired t test | 0.0144  0.0035 |

p < 0.05 ^*^, p < 0.01 ^**^, p < 0.001 ^***^, p < 0.0001 ^****^; ns – not significant

182

183

184

185

186

187

188

189

190

191

192

193

194

195

196

197
