# Supplementary material for: Long-term diet-induced obesity does not lead to learning and memory impairment in adult mice
Source: PLoS One. 2021 Sep 29;16(9):e0257921. doi: 10.1371/journal.pone.0257921 (PMC8480843; doi:10.1371/journal.pone.0257921)
Supplement: S2 Table — (DOCX) [file pone.0257921.s009.docx]

**S2 Table:** Morphological parameters of microglial cells within the hippocampus and cortex in mice after HFD and/or ND exposure for varying weeks.

Cortex

Dentate gyrus

CA1

CA3

|  | cell area [µm^2^] | cell perimeter  [µm] | convex hull area [µm^2^] | convex hull  perimeter [µm] | cell solidity | cell convexity | cell circularity | soma area [µm^2^] | soma perimeter  [µm] | soma circularity | skeleton length [µm] | skeleton branch points | skeleton end points | cell processes | branching index | critical radius | dendritic maximum |
| --- | --- | --- | --- | --- | --- | --- | --- | --- | --- | --- | --- | --- | --- | --- | --- | --- | --- |
| 8 ND | 322.31 (21.05) | 371.98  (27.24) | 1328.26  (114.49) | 147.88  (7.66) | 0.25  (0.01) | 0.43  (0.02) | 0.18  (0.01) | 40.81  (1.87) | 24.14  (0.70) | 0.94  (0.01) | 153.65  (13.29) | 9  (1) | 6  (0) | 3  (0) | 75.45  (6.35) | 9.11  (0.61) | 7  (0) |
| 4 HFD/  4 ND | 297.74  (28.89) | 334.55  (27.66) | 1200.55  (118.92) | 139.00  (7.01) | 0.26  (0.01) | 0.45  (0.02) | 0.20  (0.01) | 42.35  (1.95) | 25.03  (1.11) | 0.93  (0.02) | 136.49  (13.21) | 8  (1) | 5  (0) | 3  (0) | 61.69  (6.87) | 8.38  (0.54) | 7  (0) |
| 24 ND | 307.13  (52.12) | 353.97  (59.86) | 1271.64  (237.70) | 143.77  (12.68) | 0.25  (0.01) | 0.44  (0.04) | 0.19  (0.02) | 42.61  (2.33) | 24.97  (0.74) | 0.93  (0.01) | 146.40  (28.09) | 9  (2) | 6  (1) | 3  (0) | 67.28  (15.32) | 8.59  (0.91) | 7  (0) |
| 12 HFD/  12 ND | 377.54  (53.23) | 437.89  (73.92) | 1611.89  (291.34) | 160.23  (16.10) | 0.25  (0.01) | 0.41  (0.03) | 0.18  (0.02) | 40.30  (2.65) | 23.85  (0.71) | 0.94  (0) | 186.51  (35.67) | 12  (3) | 7  (1) | 3  (0) | 92.24  (23.22) | 10.17  (1.84) | 8  (1) |
| 24 HFD | 303.29  (45.42) | 352.03  (54.74) | 1252.93  (199.74) | 143.45  (12.35) | 0.25  (0.01) | 0.44  (0.03) | 0.19  (0.01) | 40.79  (1.99) | 24.06  (0.79) | 0.94  (0.01) | 145.72  (29.72) | 8  (3) | 6  (0) | 3  (0) | 72.00  (16.09) | 8.54  (0.81) | 7  (1) |
| 28 ND | 363.35  (55.16) | 415.35  (60.80) | 1519.79  (190.07) | 156.65  (11.17) | 0.25  (0.01) | 0.41  (0.04) | 0.18  (0.02) | 44.29  (2.06) | 25.84  (1.18) | 0.92  (0.02) | 174.50  (29.41) | 11  (2) | 6  (0) | 3  (0) | 92.87  (10.58) | 9.39  (1.36) | 7  (1) |
| 24 HFD/  4 ND | 357.28  (44.36) | 411.05  (53.26) | 1514.50  (197.43) | 156.77  (10.40) | 0.25  (0.01) | 0.42  (0.04) | 0.18  (0.02) | 42.06  (2.22) | 24.68  (1.30) | 0.93  (0.02) | 174.13  (27.01) | 11  (2) | 6  (1) | 3  (0) | 92.20  (18.20) | 10.15  (1.48) | 7  (0) |
| 8 ND | 353.66  (78.48) | 420.54  (92.17) | 1492.42  (320.96) | 155.72  (17.37) | 0.25  (0.01) | 0.41  (0.04) | 0.18  (0.02) | 41.24  (1.83) | 24.71  (0.91) | 0.92  (0.02) | 179.43  (46.48) | 11  (4) | 6  (1) | 3  (0) | 92.16  (29.18) | 10.04  (1.52) | 7  (1) |
| 4 HFD/  4 ND | 360.98  (29.24) | 435.98  (33.93) | 1630.42  (142.01) | 161.43  (6.41) | 0.24  (0.01) | 0.42  (0.02) | 0.17  (0.01) | 41.52  (3.16) | 24.65  (1.51) | 0.93  (0.02) | 182.97  (14.87) | 11  (1) | 6  (0) | 3  (0) | 89.77  (21.28) | 9.70  (1.37) | 7  (1) |
| 24 ND | 322.39  (45.50) | 394.12  (74.13) | 1472.88  (361.13) | 154.75  (18.93) | 0.24  (0.02) | 0.44  (0.03) | 0.18  (0.02) | 41.09  (2.83) | 24.37  (1.17) | 0.93  (0.01) | 163.59  (32.49) | 9  (2) | 6  (1) | 3  (0) | 88.85  (28.90) | 9.80  (2.35) | 7  (0) |
| 12 HFD/  12 ND | 361.47  (65.23) | 448.75  (84.55) | 1705.77  (346.85) | 166.89  (18.02) | 0.22  (0.02) | 0.42  (0.05) | 0.17  (0.02) | 41.05  (2.71) | 24.31  (1.03) | 0.94  (0.01) | 189.06  (40.01) | 10  (3) | 7  (1) | 3  (0) | 96.38  (17.40) | 10.94  (1.99) | 7  (1) |
| 24 HFD | 311.97  (71.04) | 376.81  (85.28) | 1400.97  (326.67) | 148.41  (20.92) | 0.24  (0.01) | 0.50  (0.15) | 0.21  (0.07) | 41.25  (4.57) | 24.13  (2.09) | 0.94  (0.03) | 157.66  (40.80) | 9  (3) | 6  (1) | 2  (0) | 79.13  (22.14) | 9.49  (1.67) | 7  (1) |
| 28 ND | 397.46  (42.36) | 482.70  (52.61) | 1781.69  (194.68) | 169.65  (11.16) | 0.24  (0) | 0.39  (0.02) | 0.16  (0.01) | 41.87  (0.38) | 24.85  (0.58) | 0.92  (0.02) | 204.34  (28.45) | 13  (2) | 7  (1) | 3  (0) | 111.54  (19.81) | 11.01  (0.84) | 7  (0) |
| 24 HFD/  4 ND | 398.82  (106.88) | 487.57  (124.43) | 1793.26  (453.78) | 169.91  (21.71) | 0.23  (0.01) | 0.39  (0.05) | 0.16  (0.02) | 42.76  (2.11) | 25.08  (0.71) | 0.93  (0) | 212.98  (62.02) | 13  (5) | 7  (1) | 3  (0) | 108.68  (41.98) | 10.71  (2.02) | 7  (1) |
| 8 ND | 279.48  (51.03) | 320.83  (49.56) | 1182.43  (179.53) | 140.28  (10.95) | 0.25  (0.01) | 0.48  (0.04) | 0.20  (0.01) | 41.11  (2.09) | 24.42  (0.68) | 0.93  (0.01) | 130.20  (26.63) | 7  (2) | 5  (1) | 3  (0) | 60.31  (12.77) | 8.95  (0.60) | 6  (1) |
| 4 HFD/  4 ND | 273.77  (54.83) | 316.30  (59.31) | 1183.19  (224.84) | 139.94  (13.03) | 0.24  (0.01) | 0.49  (0.05) | 0.20  (0.02) | 41.66  (2.25) | 24.63  (0.86) | 0.93  (0.01) | 126.30  (27.86) | 7  (2) | 5  (1) | 3  (0) | 61.18  (15.02) | 8.89  (1.18) | 6  (1) |
| 24 ND | 316.62  (56.16) | 364.87  (60.36) | 1341.85  (239.77) | 149.02  (12.48) | 0.25  (0.01) | 0.45  (0.04) | 0.19  (0.01) | 42.21  (2.65) | 24.80  (1.05) | 0.93  (0.01) | 151.24  (30.34) | 9  (2) | 6  (1) | 3  (0) | 70.56  (16.52) | 9.10  (0.97) | 7  (1) |
| 12 HFD/  12 ND | 346.16  (68.84) | 404.56  (78.44) | 1521.70  (293.52) | 158.63  (14.24) | 0.24  (0.01) | 0.43  (0.05) | 0.18  (0.02) | 41.20  (1.82) | 24.55  (0.67) | 0.93  (0.01) | 167.35  (37.66) | 9  (3) | 6  (1) | 3  (0) | 84.69  (18.79) | 10.22  (1.17) | 7  (1) |
| 24 HFD | 317.58  (70.61) | 370.00  (70.36) | 1372.68  (266.53) | 151.51  (13.55) | 0.24  (0.01) | 0.45  (0.04) | 0.19  (0.01) | 41.63  (4.29) | 24.48  (1.67) | 0.94  (0.01) | 153.18  (35.41) | 8  (3) | 6  (1) | 3  (0) | 74.50  (21.29) | 9.43  (1.08) | 7  (1) |
| 28 ND | 333.71  (64.54) | 382.04  (75.48) | 1418.63  (279.28) | 152.62  (15.35) | 0.25  (0.01) | 0.44  (0.04) | 0.18  (0.02) | 41.75  (2.53) | 24.87  (0.96) | 0.92  (0.01) | 157.15  (36.34) | 9  (3) | 6  (1) | 3  (0) | 78.47  (22.85) | 9.32  (0.89) | 7  (1) |
| 24 HFD/  4 ND | 366.03  (74.94) | 412.75  (72.35) | 1526.94  (257.78) | 159.36  (12.58) | 0.25  (0.01) | 0.42  (0.04) | 0.18  (0.01) | 42.35  (1.14) | 25.09  (0.60) | 0.93  (0.01) | 173.58  (36.28) | 10  (3) | 6  (1) | 3  (0) | 84.23  (23.11) | 9.87  (0.87) | 7  (1) |
| 8 ND | 242.11  (48.75) | 277.96  (48.19) | 955.70  (157.89) | 124.58  (9.99) | 0.26  (0.01) | 0.48  (0.04) | 0.21  (0.01) | 36.46  (3.79) | 22.23  (1.32) | 0.96  (0.01) | 109.79  (23.37) | 6  (2) | 5  (1) | 3  (0) | 52.89  (13.94) | 7.26  (0.64) | 7  (1) |
| 4 HFD/  4 ND | 238.17  (37.86) | 272.70  (44.16) | 956.39  (163.02) | 123.65  (12.16) | 0.26  (0.01) | 0.49  (0.04) | 0.22  (0.02) | 40.07  (5.12) | 23.49  (1.81) | 0.95  (0.01) | 104.66  (22.02) | 6  (2) | 5  (1) | 3  (0) | 48.36  (9.03) | 7.52  (0.49) | 6  (1) |
| 24 ND | 268.96  (48.73) | 308.44  (53.87) | 1096.68  (191.82) | 133.51  (11.48) | 0.25  (0.01) | 0.47  (0.04) | 0.20  (0.02) | 41.11  (2.20) | 24.04  (0.82) | 0.95  (0.01) | 123.44  (25.55) | 7  (2) | 5  (1) | 3  (0) | 58.81  (12.36) | 7.91  (0.66) | 7  (1) |
| 12 HFD/  12 ND | 284.95  (46.98) | 333.11  (54.39) | 1218.10  (225.25) | 141.21  (13.02) | 0.24  (0.01) | 0.46  (0.03) | 0.19  (0.02) | 41.00  (2.30) | 24.09  (0.85) | 0.94  (0.01) | 131.64  (26.45) | 7  (2) | 6  (1) | 3  (0) | 63.46  (12.54) | 8.55  (0.95) | 7  (1) |
| 24 HFD | 291.84  (52.86) | 339.03  (60.25) | 1214.72  (233.74) | 140.67  (13.47) | 0.25  (0.01) | 0.45  (0.04) | 0.19  (0.02) | 37.51  (2.44) | 22.83  (0.76) | 0.95  (0.01) | 136.28  (28.55) | 8  (2) | 5  (1) | 3  (0) | 65.97  (15.85) | 8.33  (0.94) | 7  (1) |
| 28 ND | 345.77  (84.99) | 395.80  (85.24) | 1420.38  (300.24) | 152.50  (16.03) | 0.25  (0.02) | 0.42  (0.05) | 0.18  (0.01) | 42.15  (2.88) | 24.46  (1.17) | 0.94  (0.01) | 161.35  (38.60) | 9  (3) | 6  (1) | 3  (0) | 75.86  (18.99) | 9.24  (1.17) | 7  (1) |
| 24 HFD/  4 ND | 367.47  (81.70) | 425.64  (86.02) | 1559.28  (318.44) | 158.65  (17.03) | 0.24  (0.01) | 0.41  (0.04) | 0.17  (0.02) | 41.82  (1.18) | 24.20  (0.64) | 0.95  (0.01) | 174.83  (39.58) | 10  (3) | 6  (1) | 3  (0) | 87.23  (24.50) | 9.48  (1.20) | 7  (1) |

Values are expressed as mean ± SD.
